# Hydrogen Bonding Between Ions of Like Charge in Ionic Liquids Characterized by NMR Deuteron Quadrupole Coupling Constants—Comparison with Salt Bridges and Molecular Systems

**DOI:** 10.1002/anie.201912476

**Published:** 2019-10-31

**Authors:** Alexander E. Khudozhitkov, Jan Neumann, Thomas Niemann, Dzmitry Zaitsau, Peter Stange, Dietmar Paschek, Alexander G. Stepanov, Daniil I. Kolokolov, Ralf Ludwig

**Affiliations:** ^1^ Boreskov Institute of Catalysis Siberian Branch of Russian Academy of Sciences Prospekt Akademika Lavrentieva 5 Novosibirsk 630090 Russia; ^2^ Universität Rostock Institut für Chemie Abteilung für Physikalische Chemie Dr.-Lorenz-Weg 2 18059 Rostock Germany; ^3^ Department LL&M University of Rostock Albert-Einstein-Str. 25 18059 Rostock Germany; ^4^ Leibniz-Institut für Katalyse an der Universität Rostock e.V. Albert-Einstein-Str. 29a 18059 Rostock Germany; ^5^ Novosibirsk State University Pirogova Street 2 Novosibirsk 630090 Russia

**Keywords:** DFT calculations, hydrogen bonding, ionic liquids, molecular-dynamics simulations, solid-state NMR

## Abstract

We present deuteron quadrupole coupling constants (DQCC) for hydroxyl‐functionalized ionic liquids (ILs) in the crystalline or glassy states characterizing two types of hydrogen bonding: The regular Coulomb‐enhanced hydrogen bonds between cation and anion (c–a), and the unusual hydrogen bonds between cation and cation (c–c), which are present despite repulsive Coulomb forces. We measure these sensitive probes of hydrogen bonding by means of solid‐state NMR spectroscopy. The DQCCs of (c–a) ion pairs and (c–c) H‐bonds are compared to those of salt bridges in supramolecular complexes and those present in molecular liquids. At low temperatures, the (c–c) species successfully compete with the (c–a) ion pairs and dominate the cluster populations. Equilibrium constants obtained from molecular‐dynamics (MD) simulations show van't Hoff behavior with small transition enthalpies between the differently H‐bonded species. We show that cationic‐cluster formation prevents these ILs from crystallizing. With cooling, the (c–c) hydrogen bonds persist, resulting in supercooling and glass formation.

## Graphical abstract

**Probes for like‐charge attraction**: Solid‐state NMR spectroscopy discriminates hydrogen bonding in conventional ion pairs from that between ions of like charge in ionic liquids. The measured deuteron quadrupole coupling constants for both types of ‘doubly ionic hydrogen bonds’ differ significantly. The observed cationic clusters prevent ionic liquids from crystallizing and result in supercooling and glass formation.

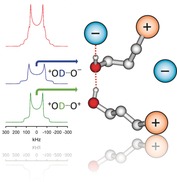

## Introduction

Salt bridges play an important role in proteins and supramolecular chemistry.^1^, ^2^, ^3^, ^4^, ^5^, ^6^, ^7^, ^8^, ^9^, ^10^, ^11^, ^12^, ^13^, ^14^ They are characterized by the sum of two types of intermolecular interaction: ionic bonding and hydrogen bonding. Salt bridges are very strong because hydrogen bonding adds to the attractive Coulomb forces between the oppositely charged residues. Typical examples of salt bridges involve the interaction of negatively charged carboxylate groups, as found, for example, in glutamic acid and aspartic acid, with positively charged ammonium groups, as present, for example, in lysine or arginine. An important example is the salt bridge between primary ammonium and carboxylate groups in biological structures, ^+^N−H⋅⋅⋅O^−^.^7^, ^8^, ^9^, ^10^, ^11^, ^12^, ^13^, ^14^ The energetics of salt bridges are typically dominated by the Coulomb interaction between the charge centers, but the total interaction remains directional due to the hydrogen bonds. Thus, salt bridges are crucial for the structure, dynamics, and function of proteins. This type of Coulomb‐enhanced hydrogen bonding is typical for ionic liquids, which consist solely of ions.^15^, ^16^, ^17^, ^18^, ^19^ So‐called “doubly ionic hydrogen bonds” (DIHB) usually result in the formation of ion pairs.^20^, ^21^, ^22^, ^23^, ^24^, ^25^ However, H‐bonds in ionic liquids are manifold. They can also be present between ions of like charge.^26^, ^27^, ^28^, ^29^, ^30^, ^31^, ^32^, ^33^, ^34^, ^35^, ^36^ This has recently been shown for cation–cation interaction by means of vibrational spectroscopy and neutron diffraction (ND).^37^, ^38^ In this case, the Coulomb forces are repulsive and need to be counter‐balanced by hydrogen bonding. For hydroxyl‐functionalized ILs, both types of ionic interaction are present in equilibrium: hydrogen bonding (O−H⋅⋅⋅O) between oppositely charged ions (c–a) and between like‐charged ions, here cations (c–c). In principle, solid‐state NMR spectroscopy allows to distinguish between (c–a) and (c–c) interactions if the proton exchange is slow on the NMR time scale.^39^, ^40^, ^41^, ^42^ Although very sensitive to the electronic environment and hydrogen bonding, deuteron quadrupole coupling constants (DQCCs) have been rarely used to characterize salt bridges in proteins, supramolecular complexes, and the related (c–a) type of interaction in ionic liquids.^43^, ^44^ DQCCs of OH groups that are involved in hydrogen bonding to like‐charged ions (c–c) are completely unknown. The main challenge here is that in the liquid phase, the proton exchange between (c–a)‐ and (c–c)‐bound species is usually fast on the NMR time scale, resulting in averaged coupling parameters and prohibiting to distinguish like‐charge attraction (c–c) from the regular ion‐pair formation (c–a). This situation may change favorably in the supercooled or glassy state of ionic liquids.

It is the purpose of this work to show that the DQCCs and the related asymmetry parameters of the electric‐field gradients provide valuable information about the strength and directionality of both types of hydrogen bonding present in hydroxyl‐functionalized ionic liquids. We find one NMR coupling parameter for ILs exhibiting (c–a) ion pairing, but two if additional (c–c) cationic‐cluster formation is present. We measure the first DQCCs describing hydrogen bonding between ions of like charge and show that they are unexpectedly smaller than those in the (c–a) ion pairs. Solid‐state NMR spectroscopy allows for counting the (c–a)‐ and (c–c)‐bound species and thus providing cluster populations. Overall, we show that cationic‐cluster formation in well‐suited ILs depends on the polarizability of the cations and the length of the hydroxyalkyl chain. If cationic‐cluster formation is present, the ILs cannot be crystallized and form glasses. The solid‐state NMR measurements are supported by density functional theory (DFT) calculations, differential scanning calorimetry (DSC) measurements, and MD simulations, which provide molecular insight into the H‐bond patterns and the delicate balance between the two types of ion pairing.

## Results and Discussion

### Synthesis and Preparation of Suitable Hydroxyl‐Functionalized ILs

We synthesized the ionic liquids 1‐(2‐hydroxyethyl)‐1‐methyl‐piperidinium bis(trifluoromethylsulfonyl)imide [HOC_2_MPip][NTf_2_] (**I**), 1‐(2‐hydroxyethyl)pyridinium bis(trifluoromethylsulfonyl)imide [HOC_2_Py][NTf_2_] (**II**), 1‐(3‐hydroxypropyl)‐1‐methyl‐piperidinium bis(trifluoromethylsulfonyl)imide [HOC_3_MPip][NTf_2_] (**III**), and 1‐(3‐hydroxybutyl)pyridinium bis(trifluoromethylsulfonyl)imide [HOC_3_Py][NTf_2_] (**IV**) using well‐established protocols (see Supporting Information). The ILs were prepared in two steps: We synthesized the OH‐functionalized onium halides, which were then used for the anion metathesis to create the desired cation/anion combinations. For the synthesis of the onium salts, we mixed equimolar amounts of the heterocyclic amine and the corresponding ω‐halide alcohol and heated the mixture up to 110 °C for 1 h. Upon cooling, the mixture started to crystallize. The crude products were recrystallized from acetone/acetonitrile mixtures to obtain the colorless crystalline product. For the metathesis of the anion (bis(trifluoromethylsulfonyl)imide, [NTf_2_]^−^), we mixed equimolar amounts of the onium halide and lithium‐bis(trifluoromethylsulfonyl)imide as aqueous solutions for 1 h. Two phases were obtained. The lower phase was washed several times with water until no residual bromine could be detected with silver nitrate solution. The resulting colorless ionic liquids were dried for several hours under vacuum at 60 °C. For detailed synthesis procedures and analytical data of each ILs see the Supporting Information.

**Scheme 1:**
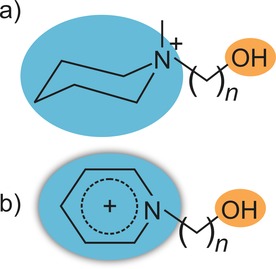
a) 1‐(*n*‐hydroxyalkyl)‐1‐methyl‐piperidinium and b) 1‐(*n*‐hydroxyalkyl)pyridinium cations as present in the ILs [HOC_*n*_Pip][NTf_2_] with *n*=2,3 (**I**, **III**) and [HOC_*n*_Py][NTf_2_] with *n*=2,3 (**II**, **IV**). The two cations differ in polarizability. Moreover, we study (c–c) cluster formation by variation of the hydroxyalkyl chain length.

As described, all ILs (**I**–**IV**) include the same [NTf_2_]^−^ counteranion and the same hydroxyl‐functional groups at the cations. This set of hydroxyl‐functionalized ILs allows studying cationic‐cluster (c–c) formation depending on the polarizability of the cation and the hydroxyalkyl chain lengths (see Scheme 1). Hydrogen/deuterium (H/D) exchange was achieved by mixing the ILs with D_2_O and removing water several times until nearly 100 % deuteration was reached as confirmed by ^1^H NMR. All samples were dried under vacuum (at 3×10^−3^ mbar) for several days and the final water concentration (<15 ppm) was checked by Karl‐Fischer titration.

### Deuteron Quadrupole Coupling Constants from Solid‐State NMR Spectroscopy

The solid‐state deuterium NMR spectrum is determined by two measurable parameters: the quadrupole coupling constant DQCC, *χ*_D_=(*e* ^2^ *q_zz_Q*/*h*), and the asymmetry parameter, *η*=(*q_xx_*−*q_yy_*)/*q_zz_*, which describes the principle elements *q* of the electric‐field gradient tensor.^45^, ^46^, ^47^ The DQCC is a measure of the magnitude of the electric‐field gradient at the deuterium site, while the asymmetry parameter provides information about the shape of the electric‐field gradient. For example, an asymmetry parameter of zero suggests a cylindrical symmetry of the electric‐field gradient tensor along the O−D bond.^47^ We determined the DQCCs from the solid‐state deuterium NMR powder patterns for ILs **I**–**IV** at 183 K (see Supporting Information).

The ^2^H NMR experiments were performed at a Larmor frequency of *ω_z_*/2π=61.42 MHz on a Bruker Avance‐400 spectrometer using a high‐power probe with a 5 mm horizontal solenoid coil. All ^2^H NMR spectra were obtained by Fourier transformation of the quadrature‐detected phase‐cycled quadrupole echo arising in the pulse sequence (90°_*x*_–*τ*_1_–90°_*y*_–*τ*_2_–acquisition–*t*), where *τ*_1_=20 μs, *τ*_2_=21 μs, and *t* is a repetition time of the sequence during the accumulation of the NMR signal. The duration of the π/2 pulse was 1.6–1.7 μs. Spectra were typically obtained with 50–20 000 scans with a repetition time ranging from 0.5 to 15 s.

**Figure 1:**
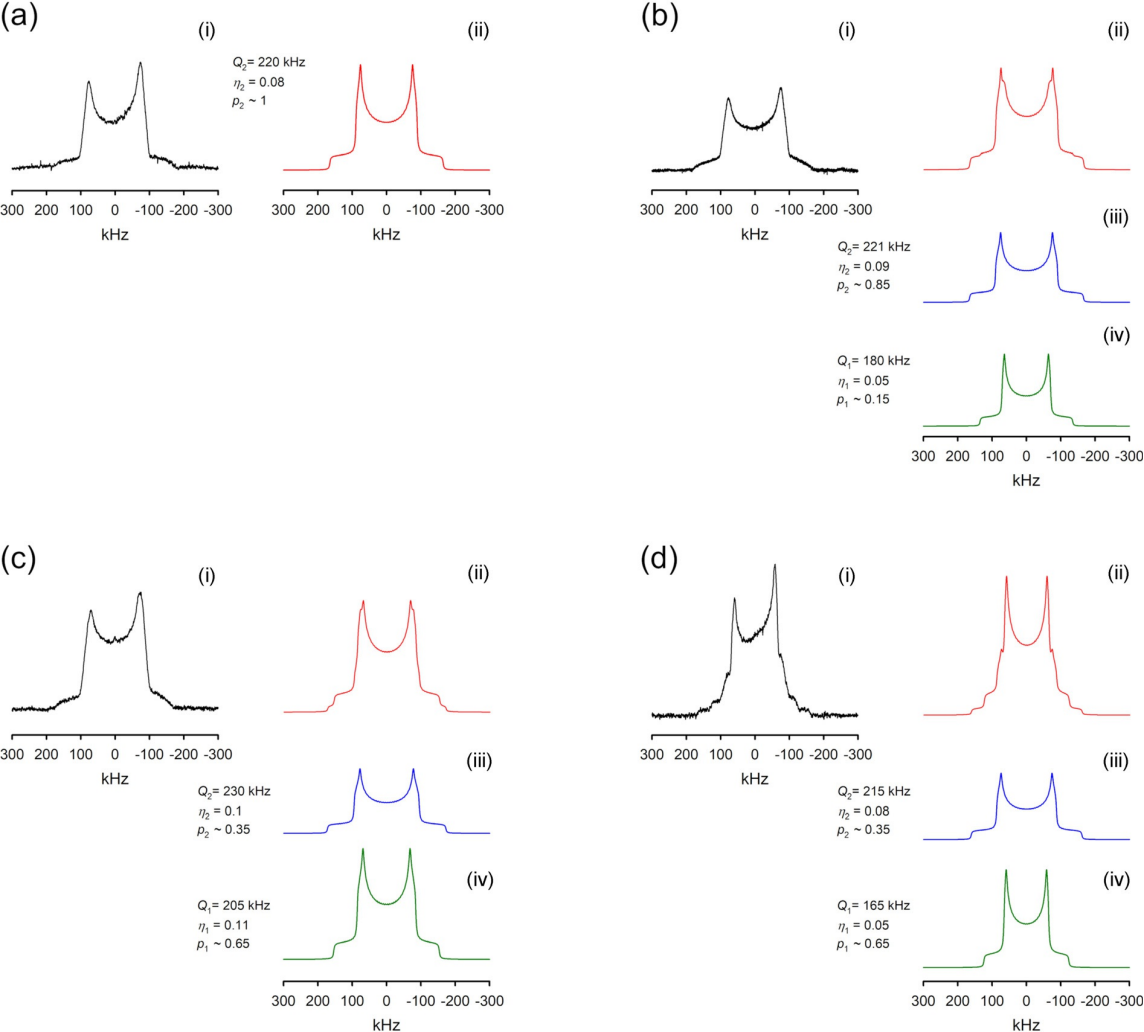
^2^H NMR spectra at 183 K and line‐shape analysis for ILs **I** [HOC_2_MPip][NTf_2_] (a), **II** [HOC_2_Py][NTf_2_] (b), **III** [HOC_3_MPip][NTf_2_] (c), and **IV** [HOC_3_Py][NTf_2_] (d). i) measured spectra, ii) simulated spectra, iii) deconvoluted (c–a) spectra, iv) deconvoluted (c–c) spectra. *Q* denotes the DQCCs, *η* the asymmetry parameters, and *p* the relative populations (fractions) of the (c–c)‐ (index 1) and (c–a)‐bound species (index 2).

All spectra show purely Pake‐powder patterns.^39^, ^40^ We obtained the DQCCs and the asymmetry parameters from a proper line‐shape analysis. The deconvoluted spectra result from a parameter optimization guided by visual inspection. The measured, analyzed, and modelled spectra are shown in Figure 1. The experimental accuracy of *χ*_D_ and *η* is ±3 kHz and ±0.01, respectively, for the dominant component, and ±5 kHz and ±0.02, respectively, for the second component of the Pake spectra. For IL **I** we observed a single Pake‐spectrum with *χ*_D_=220 kHz and *η*=0.08 (see Figure 1 a). In contrast, we could deconvolute the measured spectrum of IL **II** into two Pake patterns. One deconvoluted spectrum exhibits almost the same NMR parameters as IL **I** (*χ*_D_=221 kHz; *η*=0.09), obviously describing the same type of O−D⋅⋅⋅O interaction in both ILs. However, the second deconvoluted spectrum is clearly different and shows a smaller DQCC and asymmetry parameter, namely *χ*_D_=180 kHz and *η*=0.05 (see Figure 1 b). Smaller DQCCs suggest that the O−D⋅⋅⋅O interaction is stronger. We know from recent IR and ND experiments that (c–c) hydrogen bonds are stronger than the (c–a) ones, resulting in significant IR red‐shifts of the OH/OD stretching frequencies, lengthening of the *R*(O−H) bonds and shortening of the intermolecular *R*(O−H⋅⋅⋅O) and *R*(O⋅⋅⋅O) distances.^37^, ^38^ Thus, we conclude that the larger DQCC in both ILs of about 220 kHz can be related to the conventional (c–a) ion pairs, whereas the smaller DQCC of about 180 kHz characterizes the (c–c) interaction in cationic clusters. The smaller asymmetry parameter, 0.05 vs. 0.09, indicates that the hydrogen bond is more linear in (c–c) than in (c–a) hydrogen bonds, in accord with the above finding of stronger H‐bonds in the cationic clusters. The fact that IL **I** shows (c–a) interactions only, whereas IL **II** includes both types of H‐bond interaction, (c–a) and (c–c), is related to the different polarizabilities of the piperidinium and pyridinium cations. The pyridinium cation is highly polarizable and thus interacts favorably with the [NTf_2_]^−^ anion, leaving the hydroxyl group free to interact with other OH bonds by forming cationic clusters. In contrast, the “hard” piperidinium cation in ILs **I** and **III** interacts less favorably with the counteranion, which is then available for interacting with the OH group of the cation, resulting in typical H‐bond enhanced (c–a) ion pairs.

**Figure 2:**
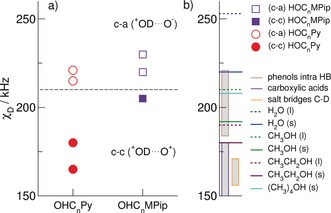
a) Measured and deconvoluted DQCCs for ILs **I**–**IV**. The DQCCs for the (c–a) hydrogen bonds above 210 kHz are given as open symbols, and the (c–c) hydrogen bonds below 210 kHz as filled symbols (with *n*=2,3). b) Comparison of the (c–c) hydrogen‐bond DQCCs with those for water, methanol, ethanol, and *tert*‐butanol in the liquid and solid phases as well as in phenols.^49^, ^50^, ^51^, ^52^, ^53^, ^54^, ^55^, ^56^, ^57^ The DQCCs for the (c–a) hydrogen bonds are related to those of salt bridges in proteins.^13^, ^43^, ^44^

If we increase the hydroxyalkyl chain length from ethyl to propyl for both types of ILs, we measure two DQCCs with *χ*_D_=230 kHz and *χ*_D_=205 kHz for IL **III**, and *χ*_D_=215 kHz and *χ*_D_=165 kHz for IL **IV**. Again, the larger values *χ*_D_=230 kHz and *χ*_D_=215 kHz can be assigned to (c–a) interactions, whereas the smaller values *χ*_D_=205 kHz and *χ*_D_=165 kHz reflect stronger (c–c) cationic interactions. The fact that both ILs form cationic clusters despite the differently favorable cations is related to the increasing distance between the positively charged ring systems and the OH functional groups within the cations. The longer tethers allow for enhanced cationic‐cluster formation (see Scheme 1). All DQCCs are shown in Figure 2, and values measured for salt bridges in supramolecular complexes or proteins are compared to (c–a) IL interactions and those measured for molecular liquids mimicking the (c–c) interaction in the ILs. Although the (c–c) DQCCs are slightly lower than those observed for solid methanol, ethanol, and *tert*‐butanol, the (c–a) DQCCs range between the values of ice and liquid water.^48^, ^49^, ^50^, ^51^, ^52^, ^53^, ^54^, ^55^, ^56^, ^57^ The (c–a) DQCCs are significantly larger than the measured values for salt bridges, which range from 156 to 171 kHz, indicating that the H‐bond‐enhanced Coulomb interaction in (c–a) ion pairs of these ILs is weak. The molecular ions have low surface charge densities, resulting in strongly attenuated Coulomb attraction and DQCCs similar to those in H‐bonded liquids. It should be noted that the (c–a) DQCCs in the hydroxyl‐functionalized ILs considered in this study are about 30 kHz higher than those measured for the protic IL triethylammonium bis(trifluoromethylsulfonyl)imide [Et_3_NH][NTf_2_] due to weaker hydrogen bonding.^58^

### Why Are (c–c) Hydrogen Bonds Stronger Than (c–a) Hydrogen Bonds?

So far, we showed that deuterons involved in (c–c) H‐bonds exhibit smaller DQCCs than those bound in (c–a) species, indicating stronger binding. These results are in accord with stronger red‐shifted IR bands and downfield‐shifted NMR proton chemical shifts for hydrogen bonding between ions of like charge as observed experimentally.^36^, ^37^, ^38^ At a first glance, it seems to be counterintuitive that the (c–c) hydrogen bonds are stronger than the (c–a) hydrogen bonds, although the first are weakened by repulsive, and the latter are enhanced by attractive Coulomb forces. To understand why hydrogen bonding is stronger in (c–c) than (c–a) clusters, we employed B3LYP‐D3/6‐31+G* calculations performed with the Gaussian 09 program and analyzed with the NBO 6.0 program.^59^, ^60^, ^61^, ^62^, ^63^, ^64^ To calculate the (c–a) and (c–c) clusters using the same method, we have used the well‐balanced but small 6‐31+G* basis set. It includes polarization as well as diffuse functions, and has been shown to be suitable for calculating hydrogen‐bonded clusters of like‐charged ions.^31^, ^32^, ^65^, ^66^ The 6‐31+G* basis set is also chosen for better comparison with earlier studies of molecular and ionic clusters.^17^, ^67^, ^68^, ^69^ We also show that the salient properties of these clusters can be robustly calculated with both smaller und larger basis sets. (see Supporting Information). Firstly, we fully optimized the cationic clusters of the IL **IV**, [HOC_3_Py][NTf_2_], up to cyclic tetramers. The calculated vibrational frequencies were all positive, showing that we calculated at least local minimum structures. Additionally, we calculated the DQCC, *χ*_D_, for each deuteron present in the (c–a) and (c–c) configurations. The DQCC describes the coupling between the nuclear quadrupole moment, *eQ*, and the principle component of the electric‐field gradient tensor, *eq_zz_*, at the deuteron nucleus. It could be shown that the relation between *χ*_D_ and *eq_zz_* is given by the equation

(1) $$\chi_{D}=\left(\frac{eQeq_{zz}}{h}\right)\left[\mathrm{kHz}\right]=2.3496eQ\left(\frac{fm^{2}}{e}\right)eq_{zz}[a.u.]$$

where the factor 2.3496 converts between the units. In principle, the DQCC can be obtained by multiplying the calculated principle component of the electric‐field gradient tensor, *eq_zz_*, of the OD hydroxyl groups in the (c–a) and (c–c) clusters of IL **IV** with a calibrated nuclear quadrupole moment, *eQ*. The calibrated *eQ* is obtained by plotting the measured gas‐phase quadrupole coupling constants from microwave spectroscopy vs. the calculated electric‐field gradients for small molecules, such as H_2_O, CH_3_OH, or formic acid, as described by Huber et al.^45^, ^46^, ^70^ The slope gives a reasonable value of *eQ*=295.5 f m^2^, which should be used for calculating DQCCs with the B3LYP‐D3/6‐31+G* method. For this set of molecules, it could also be shown that the principal axis of the deuteron electric‐field gradient is nearly axially symmetric and lies along the direction of the O−D bonds.^25^ We cannot expect that the calculated DQCCs of (c–a) and (c–c) clusters represent the measured NMR values in the crystalline or glassy state of IL **IV**. Thus, we focus on the differences of the *χ*_D_ values in (c–c) clusters relative to those obtained for the (c–a) clusters, which can be compared to Δ*χ*_D_((c–a)−(c–c)) obtained from the NMR experiment.

These spectroscopic features can be rationalized in the framework of natural bond orbital (NBO) analysis.^63^, ^64^ NBO analysis of the same (c–a) and (c–c) clusters shows a typical strong n_o_→σ*_OH_ donor–acceptor interaction with corresponding second‐order stabilization energies Δ*E* ^(2)^_*n*→σ*_ and estimated total charge transfers of *q*_CT_ for OH⋅⋅⋅O hydrogen bonds.

**Figure 3:**
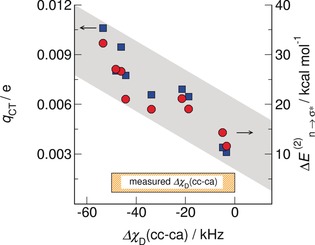
NBO second‐order stabilization energies Δ*E* ^(2)^_*n*→σ*_, (red circles, right *y*‐axis) and estimated total charge transfers *q*_CT_ (blue squares, left *y*‐axis) for (c–c) clusters with *n*=2–4 plotted vs. the calculated differences Δ*χ*_D_((c–c)−(c–a)). The almost linear dependency indicates a strong relation between NBO stabilization energies and charge transfers with the spectroscopic descriptor DQCC. The NBO parameters in particular describe the different H‐bond strength in both species. The measured Δ*χ*_D_((c‐c)−(c–a)) between (c–a) and (c–c) suggests that the glassy (c–c)‐cluster populations consist of trimers/tetramers.

The fact that the ^+^OH⋅⋅⋅OH^+^ structural motif of the (c–c) species exhibits smaller DQCCs results from significant charge transfer from the non‐bonding electron pair of the oxygen into the ^+^OH anti‐bonding orbital, leading to strong IR red‐shifts of *ν*_OH_, enhanced downfield NMR chemical shifts, *δ* ^1^H, and, in our case, smaller deuteron quadrupole coupling constants, *χ*_D_. This charge transfer is stronger than the one between cation and anion in the structural motif ^+^OH⋅⋅⋅O^−^. This is true in particular for hydrophobic anions such as [NTf_2_]^−^, where the negative charge is distributed over the entire molecule, significantly reducing the surface charge density and thus the proton‐acceptor ability of the anion. The results are summarized in Figure 3. The calculated second‐order stabilization energies, Δ*E* ^(2)^_*n*→σ*_, and the transferred charges, *q*_CT_, for the (c–c) clusters are plotted vs. the differences Δ*χ*_D_((c–c)−(c–a)) with zero indicating the average DQCC calculated for (c–a) clusters. Figure 3 shows that both NBO parameters increase almost linearly with decreasing DQCCs for (c–c) hydrogen bonding, indicated by a negative Δ*χ*_D_((c–c)−(c–a)). Obviously, both properties characterize hydrogen bonding and cooperativity in a similar way. The largest stabilization energies, Δ*E* ^(2)^_*n*→σ*_, and most intensive charge transfer, *q*_CT_, is observed for the (c–c) cyclic tetramers due to cooperative effects.^64^, ^65^, ^67^, ^68^, ^69^ Charge from the non‐bonding electron‐pair orbital of the oxygen of a first cation is donated into the OH anti‐bonding orbital of a second cation. The larger negative charge at the OH oxygen at the second cation can now be transferred into the OH anti‐bond of another cation, further enhancing hydrogen bonding. This process leads to stronger cooperativity in the (c–c) trimers and tetramers. This way, the short‐range donor–acceptor covalency forces can overcome the strong long‐range electrostatic repulsive forces, as expected for ions of like charge. The enhanced (c–c) hydrogen bonds are even stronger than those in (c–a) ion pairs despite the additional attractive Coulomb forces in the latter. Thus, cooperative stabilization energy and enhanced charge transfer lead to smaller DQCCs for (c–c) clusters. In Figure 3, we also show the experimentally measured Δ*χ*_D_((c–c)−(c–a)) for IL **IV**. A difference of 50 kHz between the (c–c) and (c–a) DQCCs suggests that the fraction of (c–c) clusters consists of significant amounts of (c–c) trimers and tetramers at least in the glassy state.

### Populations of (c–a) and (c–c) Clusters from Solid‐State NMR and MD Simulations

**Figure 4:**
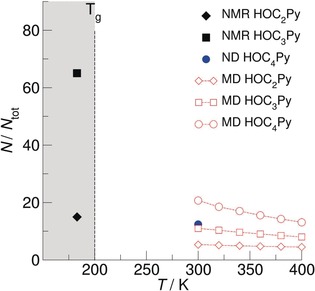
Analysis of (c–a)‐ and (c–c)‐cluster populations in [HOC_*n*_Py][NTf_2_] with *n*=2–4 from MD simulations for the liquid phase between 300 and 400 K. The filled symbols show the cluster distribution obtained from the crystalline‐ and glassy‐state NMR at 183 K (this study) and neutron diffraction (ND) experiments at 303 K.^38^

The NMR experiments in the crystalline or glassy states at 183 K allow a quantification of the populations of the local arrangements for [HOC_2_Py][NTf_2_], [HOC_3_MPip][NTf_2_], and [HOC_3_Py][NTf_2_]. In Figure 4, we show that 15 % (**II**) and 65 % (**IV**) of the cations are involved in (c–c) structural motifs. It should be noted that the experimental error for the determination of the second component is about ±5 %. Thus, the existence of components can only be claimed for relative populations larger than 10 %. For comparison, we show the populations at 303 K as recently obtained from ND measurements for [HOC_4_Py][NTf_2_] in the liquid phase.^38^ Inspired by the solid‐state NMR experiments, we also performed MD simulations using a recently improved version of the force field introduced by Köddermann et al.^71^ The refined dihedral potentials for the [NTf_2_]^−^ anion are based on extensive ab initio calculations and are leading to a better representation of the conformational space of the anion.^72^ In detail, we performed *NpT* molecular‐dynamics simulation using Gromacs 5.0.6^73^, ^74^, ^75^, ^76^, ^77^ at temperatures of 300 K, 320 K, 340 K, 360 K, 380 K, and 400 K and a pressure of *p*=1 bar. The ILs were represented by a cubic simulation box containing 512 ion pairs. The box was first equilibrated for 2 ns at *T*=500 K employing the Berendsen thermostat as well as the Berendsen barostat^78^ with coupling times of *τ*_T_=*τ*_p_=0.5 ps. After that, another equilibration run for 2 ns at the desired temperature followed. Production runs of 100 ns length utilizing the Nosé–Hoover thermostat^79^, ^80^ with *τ*_T_=1 ps and the Rahman–Parrinello barostat^81^, ^82^ with *τ*_p_=2 ps were performed for each temperature. All simulations were done with a 2.0 fs time step employing periodic boundary conditions and the LINCS algorithm^83^ for fixed bond lengths. The smooth particle‐mesh Ewald summation^84^ was applied with a mesh spacing of 0.12 nm, a real‐space cutoff 0.9 nm and fourth‐order interpolation. The relative accuracy of the Ewald sum was set to 10^−5^ corresponding to a convergence factor of *α*=3.38 nm^−1^.

**Scheme 2:**
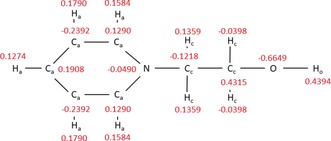
Structure of the 1‐(2‐hydroxyethyl)pyridinium [HOC_2_Py]^+^ cation with atom types and corresponding point charges *q*/*e* (red).

The force field of the [NTf_2_]^−^ anion has been published in refs. [71, 85]. The pyridinium force fields were derived from the OPLS force field for pyridine from Jorgensen et al.^86^, ^87^ The dihedral potentials of the hydroxyalkyl chain were fitted to ab initio calculations employing second‐order Møller–Plesset perturbation theory using the cc‐pVTZ basis set. The point charges were derived from the electrostatic potential according to the CHelpG scheme.^88^ The Lennard‐Jones parameters for the cations can be found in Table 1, the point charges are given in Scheme 2. Further details on the simulations are given in the Supporting Information.

**Table 1:** Lennard‐Jones parameters σ and *ϵ* for all interaction sites of the [HOC_2_Py]^+^, [HOC_3_Py]^+^, and [HOC_4_Py]^+^ cations. The assignment of the atoms is shown in Scheme 2.

site	σ [Å]	*ϵ* *k*_B_^−1^ [K]
N	3.25	85.55
Ca	3.55	35.23
Ha	2.42	15.10
Cc	3.50	33.20
Hc	2.50	15.10
Hm	2.50	15.10
O	3.12	85.60
Ho	0.00	0.00

We performed MD simulations for [HOC_2_Py][NTf_2_] and [HOC_3_Py][NTf_2_] between 300 and 400 K to show the temperature dependence of the cluster populations. Although we obtained the cluster populations from NMR (183 K) and ND (303 K) measurements only at single temperatures, and those from MD simulations only above room temperature, we can clearly conclude: a) longer hydroxyalkyl chain lengths significantly enhance the formation of (c–c) cationic clusters; b) for longer alkyl chain lengths, the polarizability of the cation is less important; c) the temperature dependence of (c–c)‐cluster formation in the liquid phase between 300 and 400 K can be described by van't Hoff plots. The ratios for the (c–c) and the (c–a) hydrogen‐bonded species vs. the inverse temperature obtained from MD‐simulation data result in transition enthalpies from (c–c) to (c–a) of about 31.24 kJ mol^−1^ (**II**), 9.42 kJ mol^−1^ (**IV**), and 3.75 kJ mol^−1^ (for [HOC_4_Py][NTf_2_]). The smaller transition enthalpy suggests that cationic clusters already exist at room temperature.

### Crystallization or Supercooling—(c–c) Cluster Formation Prevents Crystallization

**Figure 5:**
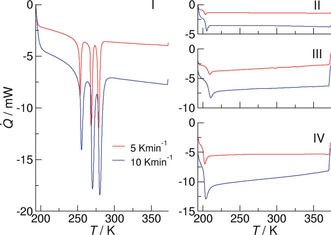
DSC traces for the ILs **I**–**IV**: For IL **I** we observe liquid/solid and several solid/solid phase transitions.^33^ For the crystalline state at 183 K, we found a single Pake pattern indicating (c–a) hydrogen‐bonded ion pairs only (see Figure 1). For ILs **II**–**IV** we observe supercooling and finally glass transition below 200 K. These ILs exhibit substantial cationic‐cluster formation characterized by two distinguished Pake patterns. Obviously, cationic‐cluster formation prevents the ILs from crystallizing.

The DSC traces of ILs **I**–**IV** as shown in Figure 5 strongly support the interpretation of the NMR spectra at low temperatures (see also the Supporting Information). Thermograms were recorded during cooling (373–193 K) and heating (193–373 K) at cooling and heating rates of 1, 5, and 10 K min^−1^. The glass‐transition temperature (*T*_g_, middle point of the heat capacity change), crystallization temperature (*T*_c_), and melting temperature (*T*_fus_) were determined from DSC thermograms during the heating scans. The summary of phase transitions is given in Table 2.

**Table 2:** Thermodynamic parameters of the observed phase transitions for ILs **I**–**IV**.

Ionic liquid	Phase transition	*T*_trs_ [K]	Δ_trs_ *H* ^o^_m_ [kJ mol^−1^]
[HOC_2_MPip][NTf_2_] (**I**)	crIII–crII	251.9±1.3	2.7±0.3
	crII–crI	266.7±1.5	4.2±0.2
	crI–liquid	276.6±0.9	4.4±0.3
			
[HOC_2_Py][NTf_2_] (**II**)	glass–liquid	200.4±0.1	–
			
[HOC_3_MPip][NTf_2_] (**III**)	glass–liquid	204.4±0.3 (1 K min^−1^) 205.5±0.1 (5 K min^−1^) 206.8±0.1 (10 K min^−1^)	–
			
[HOC_3_Py][NTf2] (**IV**)	glass—liquid	197.6±0.1 (1 K min^−1^) 199.8±0.1 (5 K min^−1^) 200.6±0.1 (10 K min^−1^)	–

During cooling from 373 to 193 K at 5 and 10 K min^−1^ cooling rates, only a heat‐capacity change corresponding to glass transitions (*T*_g_) could be observed in the DSC profiles of ILs **II** (200 K), **III** (206 K), and **IV** (200 K). The supercooled state of the (c–c) cluster‐forming ILs is obviously fairly stable. In contrast, the phase‐transition behavior is complex for IL **I**, including melting (*T*_fus_=276.2 K) and solid/solid phase transitions (*T*_ss_=266.2 K and *T*_ss_=251.6 K). For the crystalline state of IL **I** at 183 K, we observed only one Pake pattern, indicating pure (c–a) hydrogen bonding. The strong formation of cationic clusters in ILs **II**, **III** and **IV** results in supercooling and glass transition. From the combined NMR and DSC experiments, we have clear evidence that the formation of cationic clusters prevents the ILs from crystallization and liquid/solid phase transition. The resulting material is a glass.^89^ Our findings suggest that the phase behavior of this type of ILs can be controlled by cationic‐cluster formation.

## Conclusion

We measured DQCCs of hydroxyl‐functionalized ionic liquids in the crystalline and glassy states. We observed two Pake patterns for deuterons involved in normal (c–a) Coulomb‐enhanced hydrogen bonds, and in unusual (c–c) Coulomb‐weakened hydrogen bonds between cations. The DQCCs in the (c–c) cationic clusters are smaller than in (c–a) ion‐pairs, indicating stronger hydrogen bonding in accord with observed redshifts in IR spectra. These DQCCs are close to values for molecular liquids but larger than those known for salt bridges of supramolecular complexes. The (c–a) DQCCs are surprisingly large despite the additional attractive Coulomb forces. Depending on the polarizability of the cation and the alkyl chain length, the (c–c) clusters can be more strongly populated than the (c–a) ion pairs at low temperatures. The DSC traces clearly show that the ILs which form substantial amounts of (c–c) clusters do not tend to crystallize and rather form glasses.

## Conflict of interest

The authors declare no conflict of interest.

## Supporting information

As a service to our authors and readers, this journal provides supporting information supplied by the authors. Such materials are peer reviewed and may be re‐organized for online delivery, but are not copy‐edited or typeset. Technical support issues arising from supporting information (other than missing files) should be addressed to the authors.

Supplementary
