# Supplementary material for: Hydrogen Bonding Between Ions of Like Charge in Ionic Liquids Characterized by NMR Deuteron Quadrupole Coupling Constants—Comparison with Salt Bridges and Molecular Systems
Source: Angew Chem Int Ed Engl. 2019 Oct 31;58(49):17863–71. doi: 10.1002/anie.201912476 (PMC6899581; doi:10.1002/anie.201912476)
Supplement: Supplementary file 1 — Supplementary [file ANIE-58-17863-s001.pdf]

Angewandte Chemie
Eine Zeitschrift der Gesellschaft Deutscher Chemiker

Supporting Information

Hydrogen Bonding Between Ions of Like Charge in Ionic Liquids Characterized by NMR Deuteron Quadrupole Coupling Constants—Comparison with Salt Bridges and Molecular Systems

Alexander E. Khudozhitkov, Jan Neumann, Thomas Niemann, Dzmitry Zaitsau, Peter Stange, Dietmar Paschek, Alexander G. Stepanov, Daniil I. Kolokolov, and Ralf Ludwig**

anie_201912476_sm_miscellaneous_information.pdf

Contents

1. **Materials and Synthesis** **2**
2. **Sample preparation** **7**
3. **Solid State NMR** **8**
4. **Differential Scanning Calorimetry (DSC)** **9**
5. **Density functional theory (DFT) calculations on c-a and c-c clusters for [HOC$_3$Py][NTf$_2$]** **15**
6. **Molecular dynamics (MD) simulations** **23**

1 Materials and Synthesis

Sample preparation

Synthesis of the onium salts: Equimolar amounts of the heterocyclic amine and the corresponding ω-halide-alcohol were mixed and heated up to 110 °C for 1h. Upon cooling, the mixture began to crystallize. The crude product was recrystallized from acetone/acetonitrile mixtures to obtain the colorless crystalline product.
Synthesis of the bis(trifluoromethanesulfonyl)imide: Equimolar amounts of the onium halide and lithium-bis(trifluoromethanesulfonyl)imide were mixed as aqueous solutions for 1h. Two phases were obtained, the lower one was washed several times with water until no residual bromine could be detected with silver nitrate solution. The obtained colorless liquids were dried for several hours in vacuum at 60 °C.

Synthesis of the specific compounds

Apart from the reactions in aqueous solutions all reactions were performed in a moisture-guarded assembly and reflux condenser was used while heating. The used solvents were dried with molecular sieves to a water content less than 50 ppm and distilled freshly. All starting materials used in the synthesis were purchased from Sigma Aldrich and dried by conventional methods for the use in moisture-free reactions.

1-(2-Hydroxyethyl)-1-methylpiperidinium-bromide [HOC$_2$MPip][Br]

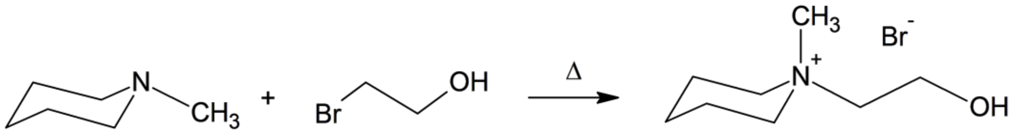

32.661 g of 2-bromoethanol (261 mmol, 19 ml) were added to a solution of 16.378 g of N-methylpiperidine (165 mmol, 20 ml) and 40 ml ethanol at room temperature. The mixture was refluxed for 2 h. After cooling to room temperature the ethanol and the residual 2-bromoethanol were removed in vacuum. The liquid crude product begins to crystallize by addition of 40 ml of acetone. The white precipitate was washed three times with dry acetone. The product was dried for 4 h in vacuum at 60 °C. (**[HOC$_2$MPip][Br]**) was obtained in 89 % yield.
EA % cal. (exp.): C 42.87 (42.65); H 8.10 (8.06); N 6.25 (6.11). ^{1}**H-NMR**(298.2 K, DMSO-d6, 300.13 MHz, [ppm]): δ = 1.48-1.58 (m, 2H, C(4)*H*2); 1.74-1.84 (m, 4H, C(2; 6)*H*2); 3.08 (s, 3H, C*H*3); 3.30-3.47 (m, 6H, C(3; 5)*H*2 +CH2−C*H*2−OH); 3.81-3.88 (m, 2H, C*H*2−CH2−OH), 5.26 (t, 1H, O*H*). ^{13}**C-NMR** (298 K, DMSO-d6, 75.46 MHz, [ppm]): δ = 20.48 (s, *C*(4)); 19.24 (s, *C*(3; 5)); 48.07 – 48.27 (m, *C*H3); 54.49 (s, *C* H2−CH2−OH); 60.77 – 60.92 (m, CH2−*C*H2−OH); 63.72 – 63.93 (m, *C*(2; 6)).

1-(2-Hydroxyethyl)-1-methylpiperidinium-bis(trifluoromethanesulfonyl)imide [HOC2MPip][NTf2]

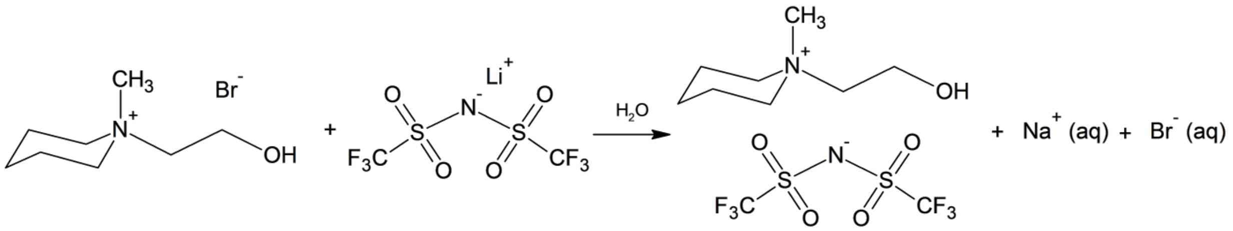

9.030 g **[HOC2MPip][Br]** (40 mmol) were solved in 10 ml H_2O and added to a solution of 11.518 g $LiNTf_2$ (40 mmol) in 10 ml H_2O. The mixture was stirred for 1h. During this time two phases were formed. The lower phase was washed several times with water until no residual bromine could be detected with silver nitrate solution. The thus obtained colorless liquid was dried for 6 h at 110 °C in vacuum. Yield: 68 %.

^{1}**H-NMR**(300 K, DMSO-d6, 250.13 MHz, [ppm]): δ = 1.46-1.57 (m, 2H, C(4)*H*2); 1.71-1.82 (m, 4H, C(2; 6)*H*2); 3.05 (s, 3H, C*H*3); 3.26-3.44 (m, 6H, C(3; 5)*H*2 CH2−C*H*2−OH); 3.78-3.86 (m, 2H, C*H*2−CH2−OH), 5.24 (t, 1H, O*H*). ^{13}**C-NMR**(298 K, DMSO-d6, 75.6 MHz, [ppm]): δ = 20.48 (s, *C*(4)); 19.24 (s, *C*(3; 5)); 48.07 – 48.27 (m, *C*H3); 54.49 (s, *C*H2−CH2−OH); 60.77 – 60.92 (m, CH2−*C*H2−OH); 63.72 – 63.93 (m, *C*(2; 6)); 119.21 (q, *C*F3). ^{19}**F-NMR**(300 K, DMSO-d6, 235.36 MHz, [ppm]): δ = −78.79 (s, C*F*3). **IR** (Transm., CaF2-Window, 12 μm- Spacer, 20 °C, 128 Scans, [cm^{-1}]): 3536 (w); 2958 (w); 2882 (w); 1846 (vw); 1797 (vw); 1471 (w); 1349 (s); 1332 (m); 1195 (s); 1138 (s); 1086 (w); 1056 (s); 986 (w).

1-(2-Hydroxyethyl)pyridinium-bromide [HOC2Py][Br]

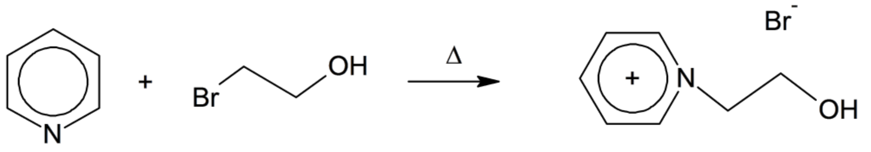

At room temperature equimolar amounts of pyridine (8.977 g; 113 mmol; 9.6 ml) and 2-bromoethanol (14.929 g; 113 mmol; 14.9 ml) were mixed and heated slowly up to 110 °C. When the reaction begins, the solution turns brown and starts to crystallize. The mixture was cooled to room temperature. The crude product was recrystallized from about 330 ml acetonitrile. The product (**[HOC2Py][Br]**) was obtained as rod-shaped colorless crystals. Yield 89 %.
EA % cal. (exp.): C 41.20 (41.22); H 4.94 (4.92); N 6.86 (6.76). ^{1}**H-NMR**(298.2 K, DMSO-d6, 300.13 MHz, [ppm]): δ = 3.86 (dd, 2H, CH2−C*H*2−OH); 4.67-4.74 (m, 2H, C*H*2−CH2−OH); 5.25(t, 1H, O*H*); 8.14-8.21 (m, 2H, *m*-C*H*); 8.60-8.67 (m, 1H, *p*-C*H*); 9.03-9.09 (m, 2H, *o*-C*H*). ^{13}**C-NMR**(298 K, DMSO-d6, 75.46 MHz, [ppm]): δ = 59.98 (s, CH2−*C*H2−OH); 63.04 (s, *C*H2−CH2−OH); 127.67 (s, *m*-*C*H); 145.15 (s, *p*-*C*H); 145.53 (s, *o*-*C*H).

1-(2-Hydroxyethyl)pyridinium-bis(trifluoromethanesulfonyl)imide [HOC₂Py][NTf₂]

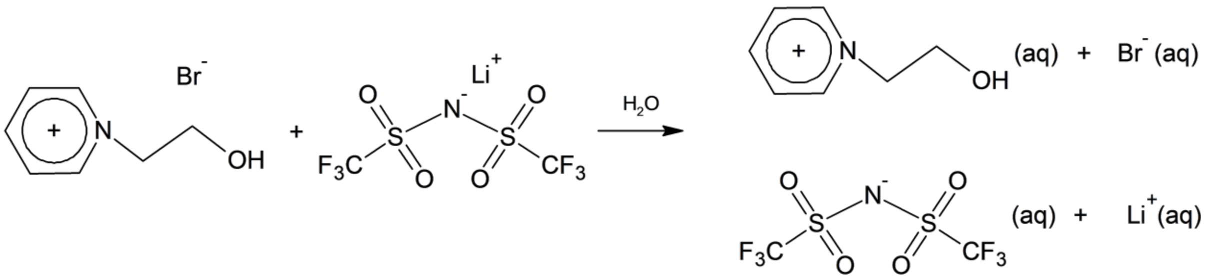

3.220 g (**[HOC$_2$Py][Br]**) (16 mmol) solved in 2 ml H_2O were added to a solution of 4.576 g lithium- bis(trifluoromethylsulfonyl)imide $LiNTf_2$ (16 mmol) in 2.5 ml H_2O. The mixture was stirred for 1 h. Two phases were obtained, the lower one was washed several times with water until no residual bromine could be detected with silver nitrate solution. The obtained colorless liquid of (**[HOC$_2$Py][NTf$_2$])** was dried for 8 h in vacuum at 60 °C. Yield 57 %.
EA % cal. (exp.): C 26.73 (26.14); H 2.49 (2.41); N 6.93 (6.54); S 15.86 (15.50).
^{1}**HNMR**(298.2 K, DMSO-d6, 300.13 MHz, [ppm]): δ = 3.88 (dd, 2H, CH2−C*H*2−OH); 4.65- 4.71 (m, 2H,
C*H*2−CH2−OH); 5.22(t, 1H, O*H*); 8.12-8.20 (m, 2H, *m*-C*H*); 8.57-8.64 (m, 1H, *p*-C*H*); 9.00-9.05 (m, 2H, *o*-C*H*). ^{13}**C-NMR**(298 K, DMSO-d6, 75.46 MHz, [ppm]): δ = 59.95 (s, CH2−*C*H2−OH); 63.01 (s, *C*H2−CH2−OH); 119.43 (q, *C*F3); 127.65 (s, *m*-*C*H); 145.12 (s, *p*-*C*H); 145.50 (s, *o*-*C*H). ^{19}**F-NMR**(298 K, DMSO-d6, 282.40 MHz, [ppm]): δ = −78.81 (s, C*F*3).
IR (Transm., CaF2-Window, 12 μm Spacer, 20 °C, 128 Scans, [cm^{-1}]): 3528 (vw); 3141 (vw); 3097 (vw); 3074 (vw); 2972 (vw); 2951 (vw); 2893 (vw); 2857 (vw); 1936 (vw); 1850 (vw); 1741 (vw); 1638 (w); 1585 (vw); 1502 (vw); 1491 (m); 1451 (vw); 1352 (vs); 1200 (s); 1136 (s); 1060 (s).

1-(3-Hydroxypropyl)-1-methylpiperidinium-chloride [HOC₃MPip][Cl]

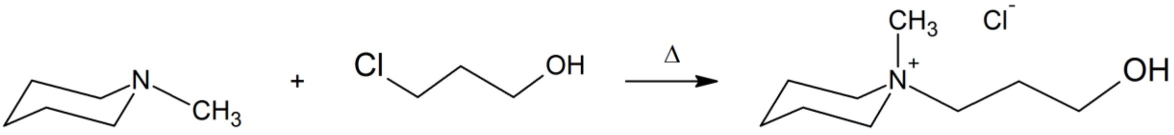

At room temperature equimolar amounts of N- methylpiperidine (8.286 g; 105 mmol; 8.9 ml) and 3-chloropropanol (14.600 g; 105 mmol; 8.8 ml) were mixed and heated slowly up to 110 °C. When the reaction begins, the solution turns yellow and starts to crystallize upon cooling to room temperature . The crude product was recrystallized from about 400 ml of an acetonitrile/acetone mixture. The product was obtained as needle-shaped colorless crystals. Yield 87 %.
^{1}**H-NMR**(298.2 K, DMSO-d6, 300.13 MHz, [ppm]): δ = 1.48-1.58 (m, 2H, C(4)*H*2); 1.71-1.88 (m, 4H, C(2; 6)*H*2 + 2H, C*H*2−CH2−OH); 3.01 (s, 3H, C*H*3); 3.28-3.43 (m, 6H, C(3; 5)*H*2 + C*H*2−CH2−CH2−OH); 3.47 (td, 2H, CH2−CH2−*CH2*−OH); 5.06 (t, 1H, O*H*). ^{13}**C-**

NMR (298 K, DMSO-d6, 75.46 MHz, [ppm]): *δ* = 19.27 (s, *C*(3; 5)); 20.58 (s, *C*(4)); 24.59 (s, *C*H2−CH2− OH); 47.07 (s, *C*H3); 57.58 (s, CH2−*C*H2−OH); 59.87 (s, *C*(2; 6)) 60.24 (s, *C*H2−CH2−CH2− OH).

1-(3-Hydroxypropyl)-1-methylpiperidinium-bis(trifluoromethanesulfonyl)imide [HOC3MPip][NTf2]

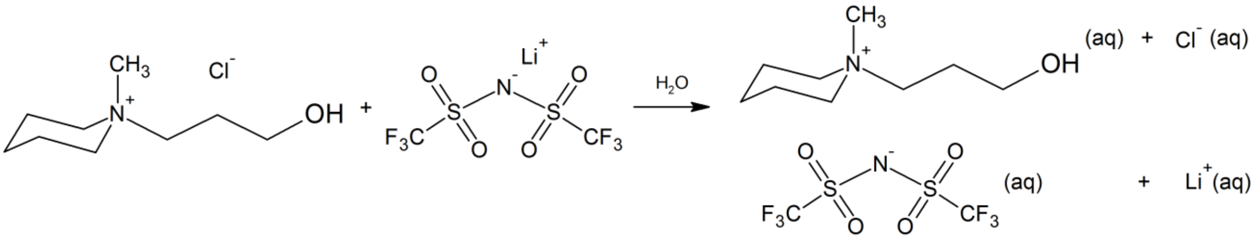

10 g (**[HOC2MPip][Cl]**) (52 mmol) were solved in 10 ml H_2O and added to a solution of 14.821g $LiNTf_2$ (52 mmol) and 10 ml H_2O. The mixture was stirred for 1h. During this time two phases were formed. The lower phase was washed several times with water until no residual chlorine could be detected with silver nitrate solution. The thus obtained colorless liquid was dried for 6 h at 110 °C in vacuum. Yield: 74 %.
^{1}**H-NMR**(298.2 K, DMSO-d6, 300.13 MHz, [ppm]): *δ* = 1.47-1.60 (m, 2H, C(4)*H*2); 1.71-1.88 (m, 4H, C(2; 6)*H*2 + 2H, C*H*2−CH2−OH); 2.98 (s, 3H, C*H*3); 3.26-3.39 (m, 6H, C(3; 5)*H*2 + C*H*2−CH2−CH2−OH); 3.49 (td, 2H, CH2−CH2−*CH2*−OH); 4.77 (t, 1H, O*H*). ^{13}**C-NMR** (298 K, DMSO-d6, 75.46 MHz, [ppm]): *δ* = 19.27 (s, *C*(3; 5)); 20.58 (s, *C*(4)); 24.59 (s, *C*H2−CH2− OH); 47.07 (s, *C*H3); 57.58 (s, CH2−*C*H2−OH); 59.87 (s, *C*(2; 6)) 60.24 (s, *C*H2−CH2−CH2 −OH); 119.44 (q, *C*F3). ^{19}**F-NMR**(300 K, DMSO-d6, 235.36 MHz, [ppm]): *δ* = −78.79 (s, C*F*3). **IR** (Transm., CaF2-Window, 12 *µ*m- Spacer, 20 °C, 128 Scans, [cm^{-1}]): 3539 (w); 2952 (w); 2882 (w); 1841 (vw); 1800 (vw); 1470 (w); 1349 (s); 1332 (m); 1194 (s); 1136 (s); 1086 (w); 1056 (s); 985 (w).

1-(3-Hydroxypropyl)pyridinium chloride [HOC2Py][Cl]

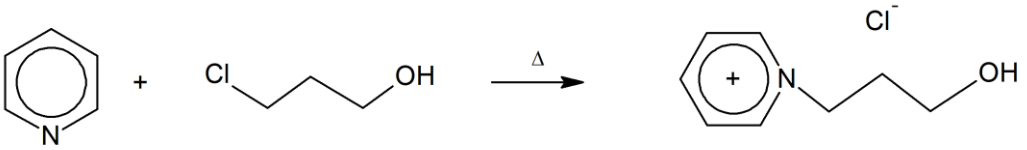

At room temperature equimolar amounts of pyridine (12.231 g; 155 mmol; 13.1 ml) and 3-chloropropanol (14.600 g; 154 mmol; 12.9 ml) were mixed and heated slowly up to 100 °C. When the reaction begins, the solution turns slightly brown and starts to crystallize upon cooling to room temperature . The crude product was recrystallized from about 450 ml acetonitrile. The product was obtained as rod-shaped colorless crystals. Yield 92 %.
^{1}**H-NMR**(298.2 K, DMSO-d_6, 300.13 MHz, [ppm]): *δ* = 2.03-2.13 (tt, 2H, CH_2−C*H*$_2$−CH_2−OH); 3.38-3.45 (dt, 2H, CH_2−CH_2− C*H*$_2$−OH); 4.76 (t, 2H, C*H*$_2$−CH_2− CH_2−OH); 5.12 (t, 1H, O*H*); 8.13-8.19 (m, 2H, *m*-C*H*); 8.58-8.65 (m, 1H, *p*-C*H*); 9.25-9.29 (m, 2H, *o*-

C*H*). [13]**C-NMR** (298 K, DMSO-d$_6$, 75.46 MHz, [ppm]): δ = 33.46 (s, CH$_2$−*C*H$_2$− CH$_2$−OH); 56.88 (s, CH$_2$−CH$_2$− *C*H$_2$−OH); 58.42 (s, *C*H$_2$−CH$_2$− CH$_2$−OH); 127.87 (s, *m*-*C*H); 145.05 (s, *p*-*C*H); 145.38 (s, *o*-*C*H). **IR** (ATR, 30°C, 128 Scans, [cm^{-1}]): 3277 (m); 3128 (w); 3093 (w); 3071 (w); 3043 (w); 3019 (w); 2969 (w); 2939 (w); 2905 (w); 2868 (w); 2804 (w); 1636 (w); 1626 (m); 1579 (vw); 1505 (w); 1484 (s); 1470(m); 1421 (w); 1379 (vw); 1360 (w); 1304 (m); 1265 (w); 1233 (w); 1174 (m); 1150 (w); 1083 (m); 1050 (s); 957 (w); 934(m); 872(vw); 819 (w); 774 (s); 6855 (vs); 638 (s).

1-(3-Hydroxypropyl)pyridinium-bis(trifluoromethanesulfonyl)imide [HOC$_3$Py][NTf$_2$]

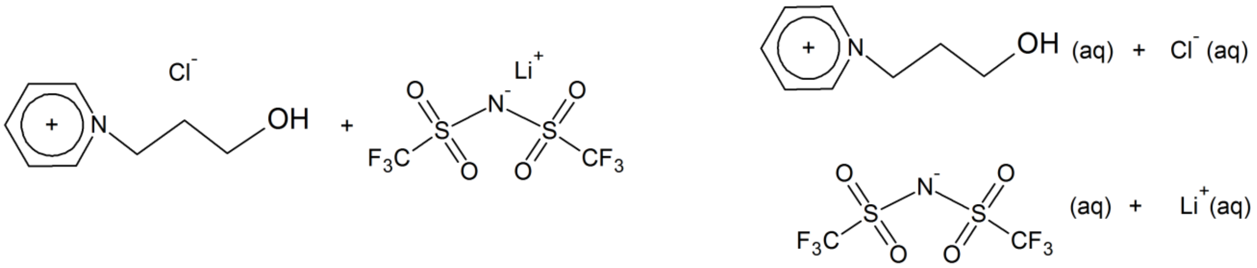

9.653 g (**[HOC$_3$Py][Cl]**) (56 mmol) solved in 10 ml H_2O were added to a solution of 16.125 g lithium- bis(trifluoromethylsulfonyl)imide LiNTf$_2$ (56 mmol) in 10 ml H_2O. The mixture was stirred for 1 h. Two phases were obtained, the lower one was washed several times with water until no residual chlorine could be detected with silver nitrate solution. The obtained colorless liquid of (**[HOC$_3$Py][NTf$_2$]**) was dried for 8 h in vacuum at 60 °C. Yield 77 %.
[1]**H-NMR**(298.2 K, DMSO-d$_6$, 300.13 MHz, [ppm]): δ = 2.03-2.13 (tt, 2H, CH$_2$−C*H*$_2$−CH$_2$−OH); 3.41-3.48 (dt, 2H, CH$_2$−CH$_2$− C*H*$_2$−OH); 4.71 (t, 2H, C*H*$_2$−CH$_2$−CH$_2$−OH); 4,88 (t, 1H, O*H*); 8.11-8.19 (m, 2H, *m*-C*H*); 8.56-8.64 (m, 1H, *p*-C*H*); 9.10-9.17 (m, 2H, *o*-C*H*). [13]**C-NMR**(298 K, DMSO-d$_6$, 75.46 MHz, [ppm]): δ = 33.27 (s, CH$_2$−*C*H$_2$−CH$_2$−OH); 57.04 (s, CH$_2$−CH$_2$− *C*H$_2$−OH); 58.59 (s, *C*H$_2$−CH$_2$− CH$_2$−OH); 119.43 (q, *C*F$_3$); 127.87 (s, *m*-*C*H); 144.99 (s, *p*-*C*H); 145.37 (s, *o*-*C*H). [19]**F-NMR**(298 K, DMSO-d$_6$, 282.40 MHz, [ppm]): δ = −78.81 (s, C*F*$_3$). **IR** (Transm., CaF$_2$-Window, 12 μm Spacer, 20 °C, 128 Scans, [cm^{-1}]): 3552 (w); 3299 (m); 3139 (w); 3097 (w); 3071 (w); 2943 (w); 2881 (w); 1637 (m); 1491 (m); 1318 (w); 1286 (w); 1221 (w); 1170 (w); 1060 (vs); 953 (w).

2 Sample preparation

In order to prepare the samples for the NMR experiments, the deuterated ionic liquids $[DOC_2MPip]NTf_2$, $[DOC_2Py]NTf_2$, $[DOC_3MPip]NTf_2$, and $[DOC_3Py]NTf_2$, were loaded into a glass tube (5 mm o.d.; 20 mm long), connected to a high vacuum grade valve (HI-VAC). All manipulations were performed in argon atmosphere. The samples were then attached to a vacuum line and the argon was pumped off under vacuum to a final pressure above the samples of 10^{-2} Pa. To fully degas the material the samples were slowly introduced into liquid nitrogen 2-3 times, while being connected to vacuum line. After degassing, the necks of the tubes were sealed off, while the material samples were maintained in liquid nitrogen in order to prevent their heating by the flame. The sealed samples were then transferred into an NMR probe for analysis with ^2H NMR spectroscopy.

3 Solid State NMR

In order to prepare sample for the NMR experiments, the OH-deuterated ionic liquid was loaded into a glass tube (5 mm o.d.; 20 mm long), connected to a high vacuum grade valve (HI-VAC). All manipulations were performed in argon atmosphere. The sample was then attached to a vacuum line and the argon was pumped off under vacuum to a final pressure above the sample of 10^{-2} Pa. To fully degas the material, the sample was slowly introduced into liquid nitrogen 2-3 times, while being connected to vacuum line. After degassing, the neck of the tube was sealed off, while the material sample was maintained in liquid nitrogen in order to prevent its heating by the flame. The sealed sample was then transferred into an NMR probe for analysis with ^{2}H NMR spectroscopy. The temperature of the samples was controlled with a flow of nitrogen gas by a variable-temperature unit BVT-3000 with a precision of about 1 K.

It should be noted that in each the NMR experiments were conducted by first cooling the sample in the liquid nitrogen, rapidly transferring the sample in the pre-cooled probe and then heating it up to the required temperature. Such procedure allowed a perfect reproducibility of the experimental results in the solid phase.

^{2}H NMR experiments were performed at Larmor frequency $\omega_z / 2\pi = 61.42$ MHz on a Bruker Avance-400 spectrometer, using a high power probe with 5 mm horizontal solenoid coil. All ^{2}H NMR spectra were obtained by Fourier transformation of quadrature-detected phase-cycled quadrupole echo arising in the pulse sequence ($90°_x - \tau_1 - 90°_y - \tau_2$ – acquisition – t), where τ_1 = 20 μs, τ_2 = 21 μs and t is a repetition time of the sequence during the accumulation of the NMR signal. The duration of the $\pi/2$ pulse was 1.6-1.7 μs. Spectra were typically obtained with 50 - 20000 scans with repetition time ranging from 0.5 to 15 seconds.

4. DSC

Differential scanning calorimetry (DSC) measurements were carried out by using a Mettler Toledo DSC 822e with a Huber TC100MT cooler under N_2 atmosphere. The samples for DSC measurements were tightly sealed in Al pans of 40 μl volume. All handling operations with sample were carried out in a glove-box under a nitrogen atmosphere (residual concentrations of oxygen 1.0 ppm and water 0.3 ppm). Pans and samples were weighted with Sartorius MSE3.6P-000-DM microbalances with the standard uncertainty of $5 \cdot 10^{-6}$ g. The calibration of Mettler Toledo DSC 822e was checked with melting behavior of the reference samples of indium, *n*-octane, and twice distilled water. The temperature of fusion agreed with recommended value better than 0.3 K and the fusion enthalpy within 0.2 kJ·mol^{-1}.

Thermograms were recorded during cooling (373-193 K) and heating (193-373 K) at cooling and heating rates of 1, 5, and 10 K· min^{-1}. The glass transition temperature (T_g, middle point of the heat capacity change), crystallization temperature (T_c), and melting temperature (T_{fus}) were determined from DSC thermograms during the heating scans. The summary of phase transitions is given in Table S1.

[HOC_2MPip][NTf_2] (I) [1]

For this IL the crystallization of the sample was observed on cooling. Changing the heating and cooling rates doesn't change the temperature of the observed phase transitions. That obviously shows the thermodynamic nature of the recorded transitions.

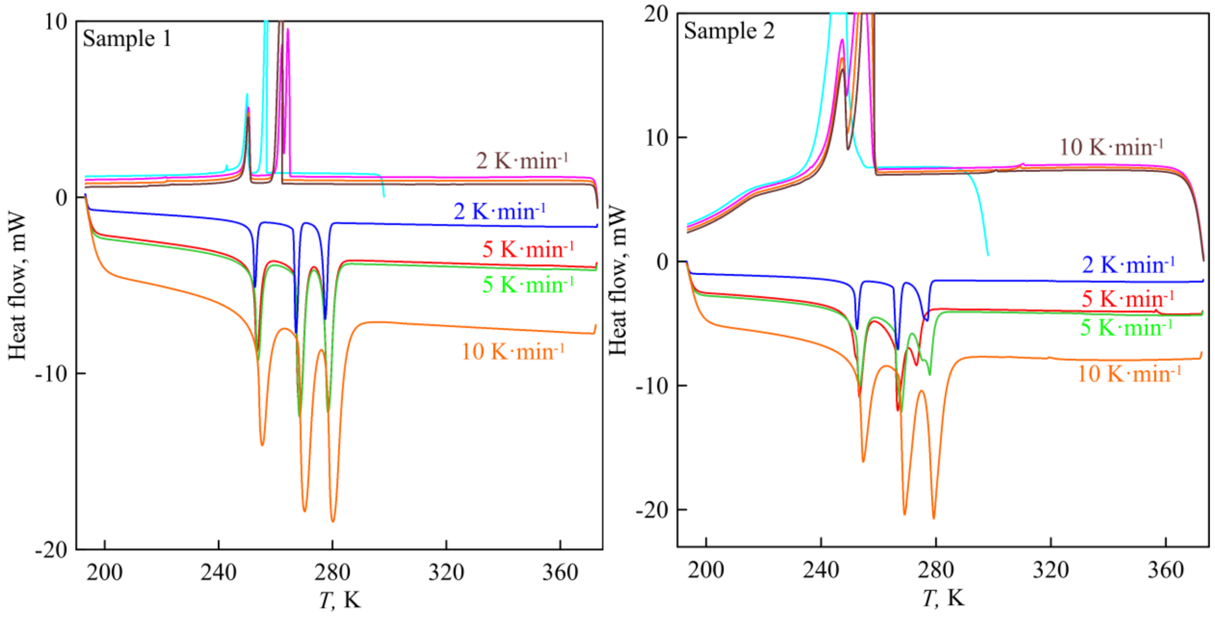

SI Fig. S1 The DSC profile for [HOC_2MPip][NTf_2] (**II**) samples; blue line is heating with 2 K·min^{-1}, green and read lines – heating with 5 K·min^{-1}, orange line - heating with 10 K·min^{-1}. The curves with the same heating or cooling rate are shifted for 0.1 mW for better illustration.

[HOC_2Py][NTf_2] (II) [1]

No phase transition was observed during cooling down to 193 K. During heating the glass transition at 200.4 K was observed and reproduced for both samples. No fusion or crystallization peak has been seen for both samples at heating rates from 2 to 10 K·min^{-1}.

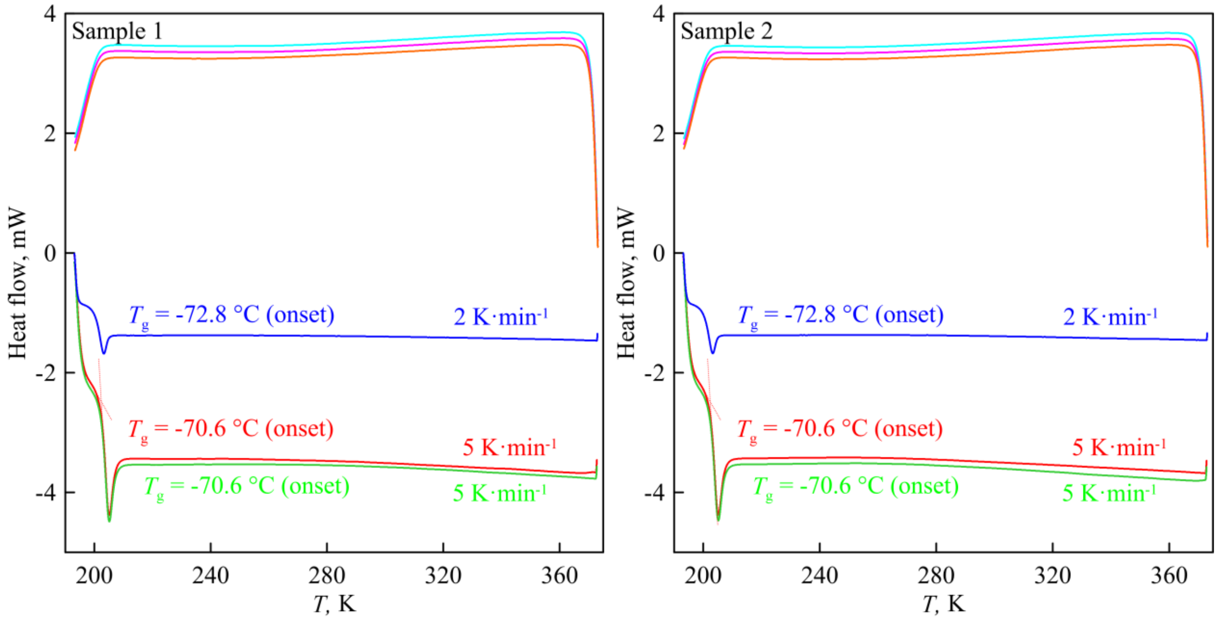

SI Fig. S2 The DSC profile for [HOC_2Py][NTf_2] (**II**) samples; blue line is the heating with 2 K·min^{-1}, green and red lines – heating with 5 K·min^{-1}. No peak of fusion was observed. The curves with the same heating or cooling rate are shifted for 0.1 mW for better illustration.

[HOC_3Pip][NTf_2] (III), [HOC_3Py][NTf_2] (IV)

For all these samples no crystallization has been during cooling and heating runs. According to Beaman−Kauzmann rule one can expect $T_g \approx 2/3\ T_{fus}$ and evaluate the fusion temperature for studied ILs. As a matter of fact, the highest rate of crystallization can be obtained at temperatures 20 - 30 K lower than T_{fus}. We have annealed the samples at these temperatures to obtain the crystal phase of the studied compounds.

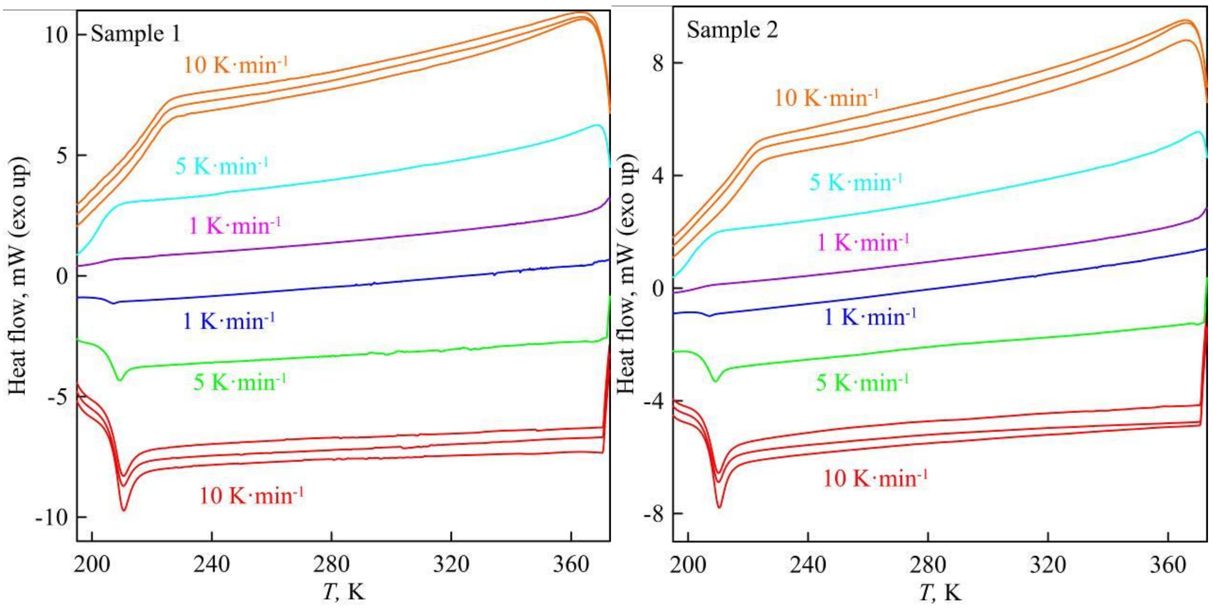

SI Fig. S3 The DSC profile for [HOC_3MPip][NTf_2](**III**) samples; blue line is the heating with 1 K·min^{-1}, green and red lines – heating with 5 and 10 K·min^{-1}, correspondingly. The cooling curves are marked as follows: magenta – 1 K·min^{-1}; cyan - 5 K·min^{-1}, and orange - 10 K·min^{-1}. The curves with the same heating or cooling rate are shifted for 0.2 mW for better illustration.

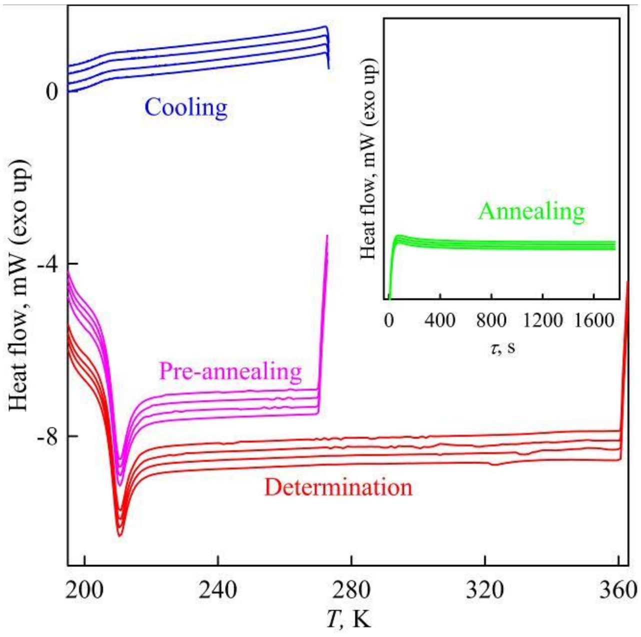

SI Fig. S4 The DSC profile for [HOC_3MPip][NTf_2](**III**) samples with annealing procedure; blue lines are the cooling after annealing, green lines show the heat flow during annealing, magenta corresponds to heating before annealing and red lines correspond to heating with 10 K·min^{-1} after annealing. The curves with the same heating or cooling rate are shifted for 0.3 mW for better illustration.

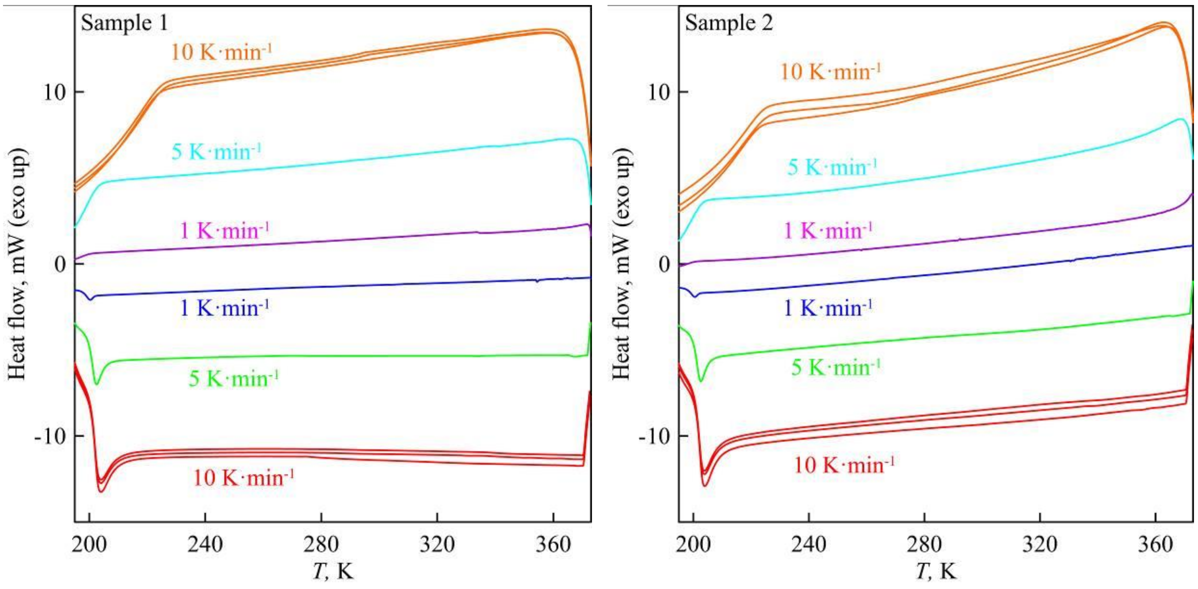

SI Fig. S5 The DSC profile for [HOC$_3$Py][NTf$_2$](**IV**) samples; blue line is the heating with 1 K·min^{-1}, green and red lines – heating with 5 and 10 K·min^{-1}, correspondingly. The cooling curves are marked as follows: magenta – 1 K·min^{-1}; cyan - 5 K·min^{-1}, and orange - 10 K·min^{-1}. The curves with the same heating or cooling rate are shifted for 0.2 mW for better illustration.

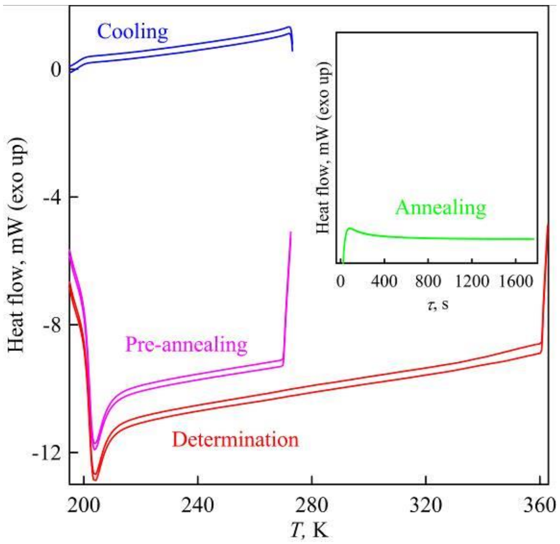

SI Fig. S6 The DSC profile for [HOC$_3$Py][NTf$_2$](**IV**) samples with annealing procedure; blue lines are the cooling after annealing, green lines show the heat flow during annealing procedure, magenta corresponds to heating before annealing and red lines correspond to heating with 10 K·min^{-1} after annealing. The curves with the same heating or cooling rate are shifted for 0.3 mW for better illustration.

Table S1. The thermodynamic parameters of observed phase transitions for studied ILs.

Ionic liquid	Phase transition	T_{trs}, K	$\Delta_{trs}H^{o}_{m}$, kJ·mol^{-1}	Comments
$[HOC_2MPip][NTf_2]$ (I)	crIII-crII	251.9 ± 1.3	2.7 ± 0.3	Ref. 1
	crII-crI	266.7 ± 1.5	4.2 ± 0.2	
	crI – liquid	276.6 ± 0.9	4.4 ± 0.3	
$[HOPy][NTf_2]$ (**II**)	glass - liquid	200.4 ± 0.1	-	Ref. 1
$[HOC_3MPip][NTf_2]$ (**III**)	glass-liquid	204.4 ± 0.3 (1 K·min^{-1})	-	
		205.5 ± 0.1 (5 K·min^{-1})		
		206.8 ± 0.1 (10 K·min^{-1})		
[HOC3Py][NTf2] (**IV**)	glass-liquid	197.6 ± 0.1 (1 K·min-1)	-	
		199.8 ± 0.1 (5 K·min-1)		
		200.6 ± 0.1 (10 K·min-1)		

References:

[1] T. Niemann, D. Zaitsau, A. Strate, A. Villinger, R. Ludwig, *Sci. Rep.* **2018**, *8*, 14753.

7. Density functional theory (DFT) calculations on [HOC_3Py][NTf_2] clusters

***** pyridinium_C3_NTf2_b3lyp_6-31+Gp_monomer_D3.g09 (c-a)**

6	0	4.434121	-0.291884	-0.470492
7	0	3.384067	0.551822	-0.596495
6	0	2.290456	0.195540	-1.304270
6	0	2.208278	-1.055110	-1.899906
6	0	3.274018	-1.941416	-1.769207
6	0	4.410136	-1.543471	-1.055564
6	0	3.447541	1.677646	1.667366
6	0	2.293102	0.870967	2.258689
8	0	2.421148	-0.476511	1.812394
9	0	-3.519859	1.189383	-0.533543
6	0	-2.481299	2.006069	-0.750612
9	0	-2.776216	3.223510	-0.272091
16	0	-0.941296	1.359715	0.126700
7	0	-0.761637	-0.074522	-0.612246
16	0	-0.760947	-1.507454	0.135273
6	0	-2.572174	-1.959505	0.422224
9	0	-2.640934	-3.188270	0.953422
9	0	-2.268025	2.098683	-2.070565
8	0	0.132221	2.259409	-0.350353
8	0	-1.257195	1.349967	1.556871
8	0	-0.281744	-2.495537	-0.837910
8	0	-0.180072	-1.502572	1.492111
9	0	-3.146383	-1.087859	1.258153
9	0	-3.236282	-1.951075	-0.742127
1	0	1.290617	-1.329364	-2.404461
1	0	1.544577	-0.910920	1.841940
1	0	3.438747	2.689276	2.092783
1	0	4.397398	1.216634	1.968115
1	0	2.348020	0.923200	3.356613
1	0	1.330781	1.299890	1.955675
1	0	5.261563	-2.203019	-0.929192
1	0	3.216636	-2.934377	-2.204197
1	0	1.481312	0.913256	-1.338736
1	0	5.265481	0.061199	0.126397
6	0	3.392562	1.852034	0.142505
1	0	2.480523	2.376144	-0.144998
1	0	4.257211	2.418195	-0.218849

***** pyridinium_C3_NTf2_cation_cation_b3lyp_6-31+Gp_dimer_D3.g09 (c-c)**

6	0	-0.065823	-3.195163	0.135749
7	0	-1.410858	-3.037518	0.041377

6	0	-2.073852	-2.225734	0.895003
6	0	-1.392217	-1.533799	1.885769
6	0	-0.013810	-1.695709	2.006322
6	0	0.656267	-2.543303	1.115920
6	0	-1.882137	-3.221561	-2.444608
6	0	-2.585546	-1.906427	-2.761109
8	0	-2.071286	-0.912999	-1.872608
8	0	2.908703	-1.561278	3.230295
16	0	3.910264	-0.871775	2.407915
8	0	5.313002	-0.817332	2.820311
6	0	3.327011	0.921773	2.471780
9	0	3.998721	1.680709	1.571145
7	0	3.640506	-1.199765	0.846870
16	0	4.811484	-1.612581	-0.190308
8	0	5.278592	-0.465401	-1.003195
9	0	2.014065	1.014924	2.189034
9	0	3.538971	1.433415	3.688181
8	0	5.800621	-2.580029	0.280345
6	0	3.690300	-2.555099	-1.380679
9	0	2.706142	-1.771248	-1.851815
9	0	4.420817	-2.995010	-2.413110
9	0	3.126435	-3.617947	-0.772546
8	0	0.380675	0.305527	-2.157576
6	0	0.654691	0.755108	-0.834603
6	0	1.575912	1.968163	-0.934067
7	0	3.817094	2.705253	-1.771552
6	0	3.512398	3.869223	-2.394064
6	0	4.398022	4.932156	-2.385431
6	0	5.621698	4.793483	-1.719959
6	0	5.919489	3.585273	-1.092858
6	0	4.997279	2.546075	-1.131985
9	0	-7.169000	1.071558	0.256295
6	0	-6.920264	-0.241801	0.183607
9	0	-7.780400	-0.801179	-0.680030
16	0	-5.160446	-0.549167	-0.422343
7	0	-4.281933	0.149207	0.746713
16	0	-3.295760	1.412844	0.534013
6	0	-4.371850	2.949380	0.741537
9	0	-3.585626	4.037973	0.750807
9	0	-7.099920	-0.787669	1.392432
8	0	-5.007371	-2.013459	-0.306602
8	0	-5.079276	0.048296	-1.762536
8	0	-2.378651	1.452565	1.677448
8	0	-2.735460	1.555620	-0.823714
9	0	-5.239985	3.051410	-0.272437
9	0	-5.050380	2.894882	1.894947
1	0	-1.951839	-0.858046	2.520853
1	0	-2.626812	-0.107103	-1.869657
1	0	-2.236351	-3.982840	-3.151228
1	0	-0.801645	-3.115737	-2.607640
1	0	-2.392988	-1.625784	-3.806538

1	0	-3.667638	-2.005168	-2.622906
1	0	-0.467625	-0.187789	-2.139940
1	0	1.156201	-0.032549	-0.253939
1	0	-0.264899	1.041507	-0.311289
1	0	1.029151	2.794893	-1.404330
1	0	1.878947	2.282270	0.069252
1	0	5.177889	1.570603	-0.692894
1	0	6.330231	5.616002	-1.697887
1	0	4.128264	5.851585	-2.893319
1	0	1.728776	-2.672933	1.175070
1	0	0.553856	-1.176784	2.770577
1	0	-3.142095	-2.141862	0.750759
1	0	0.396715	-3.852358	-0.590969
1	0	6.857754	3.427821	-0.572419
1	0	2.547744	3.907115	-2.886343
6	0	-2.155990	-3.759594	-1.031179
1	0	-3.217297	-3.679262	-0.789571
1	0	-1.859349	-4.810141	-0.956765
6	0	2.808168	1.599652	-1.762833
1	0	3.311846	0.724593	-1.359221
1	0	2.535429	1.391584	-2.799303

***** pyridinium_C3_NTf2_cation_cation_b3lyp_6-31+Gp_trimer_D3.g09 (c-c)**

6	0	-0.342511	0.143526	3.215786
7	0	-1.643486	0.431472	2.966927
6	0	-2.569121	-0.555227	2.868318
6	0	-2.195893	-1.884711	2.991795
6	0	-0.853494	-2.201695	3.216589
6	0	0.078687	-1.168991	3.343703
6	0	-2.078415	2.056753	1.154194
6	0	-2.294929	3.525211	0.785099
8	0	-2.129544	3.714588	-0.628663
8	0	-2.125435	1.659582	-2.405797
6	0	-3.212776	1.569414	-3.331536
6	0	-3.271408	0.157243	-3.911136
7	0	-2.306551	-1.393390	-2.168368
6	0	-1.958179	-0.793616	-1.012235
6	0	-0.792423	-1.147101	-0.356599
6	0	0.011729	-2.156325	-0.877293
6	0	-0.379628	-2.789330	-2.063015
6	0	-1.544047	-2.386213	-2.689304
8	0	0.253906	1.763747	-3.836049
6	0	0.692216	0.431479	-3.624968
6	0	1.846360	0.341158	-2.623051
7	0	4.238833	1.032598	-2.243780
6	0	4.830505	2.168524	-1.810298
6	0	5.937416	2.109938	-0.976833
6	0	6.431509	0.867980	-0.577542

6	0	5.806877	-0.295010	-1.034923
6	0	4.711768	-0.182720	-1.874532
8	0	2.590138	4.441598	-2.254501
16	0	1.924139	4.378738	-0.949078
7	0	2.372471	2.980303	-0.277245
16	0	1.737860	2.328670	1.050116
8	0	0.782696	3.142893	1.820309
6	0	2.786475	5.723997	0.057896
9	0	2.308076	5.769099	1.305150
9	0	4.106637	5.463429	0.106690
9	0	2.600687	6.909500	-0.530647
8	0	0.494511	4.703272	-0.838698
8	0	1.387934	0.922933	0.794895
6	0	3.258885	2.232587	2.159125
9	0	4.237171	1.521044	1.583005
9	0	3.721617	3.458199	2.431756
9	0	2.906826	1.635933	3.312599
8	0	1.854219	-3.649567	2.561290
16	0	3.102845	-3.676703	1.786491
8	0	4.100243	-4.719159	2.017333
6	0	3.930330	-2.056709	2.292978
9	0	5.097320	-1.868596	1.660419
7	0	2.686607	-3.399784	0.240013
16	0	3.623506	-3.631592	-1.049006
8	0	2.963311	-2.929496	-2.170093
9	0	3.115377	-1.026588	2.000238
9	0	4.153031	-2.058422	3.617131
8	0	5.068618	-3.436390	-0.869608
6	0	3.392332	-5.452882	-1.493358
9	0	2.084809	-5.697205	-1.703425
9	0	4.065315	-5.720268	-2.622109
9	0	3.831148	-6.248044	-0.518583
9	0	-4.881615	2.446118	-0.645401
6	0	-6.097485	1.980757	-0.298304
9	0	-6.828189	3.000043	0.163668
9	0	-6.688167	1.475904	-1.390147
16	0	-5.947061	0.681188	1.058437
7	0	-5.096194	-0.405399	0.175335
16	0	-5.074771	-1.970581	0.579747
6	0	-6.600018	-2.742897	-0.230955
9	0	-6.521704	-4.076655	-0.116785
8	0	-5.099960	1.324884	2.082138
8	0	-7.314831	0.300225	1.405800
8	0	-3.964320	-2.591256	-0.158004
8	0	-5.207808	-2.232026	2.020867
9	0	-7.730012	-2.319132	0.328505
9	0	-6.611226	-2.423849	-1.541273
1	0	1.127297	-1.382635	3.503675
1	0	-0.510033	-0.625893	0.547270
1	0	-2.248358	2.421081	-1.783126
1	0	-4.092115	0.129865	-4.639168

1	0	-2.349491	-0.058490	-4.466034
1	0	-3.077178	2.292975	-4.147717
1	0	-4.155705	1.800664	-2.823160
1	0	-0.546237	1.897486	-3.284232
1	0	1.010188	0.023649	-4.595848
1	0	-0.131432	-0.193305	-3.263275
1	0	1.544190	0.759298	-1.659150
1	0	2.087442	-0.715792	-2.467906
1	0	-1.234515	4.075355	-0.786106
1	0	-3.311926	3.838854	1.039993
1	0	-1.581489	4.160589	1.320939
1	0	-1.132672	1.724369	0.719281
1	0	-2.890041	1.458174	0.734065
1	0	4.187614	-1.049257	-2.258654
1	0	7.285365	0.804341	0.090330
1	0	6.380986	3.038067	-0.633612
1	0	0.329672	0.987886	3.274846
1	0	-0.517927	-3.231833	3.275833
1	0	-3.587149	-0.238415	2.677445
1	0	0.229436	-3.573470	-2.498397
1	0	0.926609	-2.458216	-0.375288
1	0	-2.640214	-0.044976	-0.648500
1	0	-2.964399	-2.641214	2.885094
1	0	-1.900536	-2.825629	-3.613671
1	0	6.132184	-1.288646	-0.748898
1	0	4.381459	3.098520	-2.134561
6	0	3.054523	1.109802	-3.155353
1	0	3.380821	0.709793	-4.122094
1	0	2.806785	2.164600	-3.276067
6	0	-3.538492	-0.953237	-2.879648
1	0	-4.250230	-0.634599	-2.116022
1	0	-3.949478	-1.844307	-3.359283
6	0	-2.038872	1.840530	2.668792
1	0	-1.294002	2.486741	3.135270
1	0	-3.015805	2.013614	3.122013

***** pyridinium_C3_NTf2_b3lyp_6-31+Gp_cation_cation_tetramer_D3.g09 (c-c)**

C	-5.538088	-3.197406	-1.003359
N	-4.545942	-2.943787	-1.883581
C	-3.840967	-3.945630	-2.461119
C	-4.135073	-5.266092	-2.169466
C	-5.176240	-5.553124	-1.277702
C	-5.884949	-4.504196	-0.695002
C	-4.194372	-1.524276	-2.168963
C	-3.158852	-0.994397	-1.181711
C	-1.777378	-1.603686	-1.328125
O	-1.339617	-1.446506	-2.691665
O	1.193661	-1.487385	-2.088820
C	1.703521	-2.680153	-1.495917

C	1.642245	-3.869797	-2.455813
C	1.918746	-5.224356	-1.781874
N	3.182202	-5.257726	-0.986590
C	4.361702	-5.532760	-1.595819
C	5.540265	-5.556654	-0.872205
C	5.512253	-5.259402	0.493242
C	4.292859	-4.958530	1.096824
C	3.136446	-4.967549	0.335701
O	-0.801861	1.185851	-2.792174
C	-1.879776	1.935861	-3.366595
C	-1.868110	3.332102	-2.759816
C	-2.328086	3.450178	-1.299477
N	-1.610693	2.560439	-0.335848
C	-0.262161	2.545076	-0.293331
C	0.411014	1.736479	0.604677
C	-0.314511	0.933559	1.485585
C	-1.707989	0.990910	1.449857
C	-2.332134	1.817252	0.529395
O	1.809976	0.673864	-3.622317
C	2.796265	1.692183	-3.483095
C	3.109771	1.990739	-2.010570
C	4.246421	3.004866	-1.878055
N	4.485895	3.397561	-0.453353
C	3.944545	4.540801	0.028941
C	4.127032	4.914017	1.349974
C	4.862589	4.091021	2.201111
C	5.402551	2.909574	1.689963
C	5.207092	2.583388	0.356929
O	-1.288994	4.750411	1.519190
S	-0.223009	5.745723	1.706233
O	-0.573648	7.109059	2.099666
N	0.961758	5.639311	0.568512
S	0.623917	5.954487	-0.970358
C	1.491811	7.607343	-1.245834
F	2.821670	7.459222	-1.049724
O	1.381582	5.021433	-1.842025
O	-0.792639	6.191601	-1.278500
C	0.818326	5.059956	3.123076
F	1.883312	5.842805	3.375001
F	0.056763	5.012337	4.218881
F	1.261281	3.817505	2.849520
F	1.292423	8.024447	-2.500944
F	1.035874	8.524401	-0.393587
O	2.361950	-4.015058	3.124404
S	1.269120	-3.805500	4.092968
O	1.264536	-4.527092	5.360263
N	-0.210877	-3.887606	3.444244
S	-0.504296	-3.557030	1.902531
O	-0.028018	-4.572377	0.932356
C	-2.366524	-3.831412	2.012636
F	-2.927838	-2.998760	2.892571

F	-2.663553	-5.093948	2.331931
F	-2.897869	-3.571616	0.790813
O	-0.346965	-2.151474	1.483995
C	1.458713	-1.989061	4.594202
F	1.481709	-1.183037	3.515238
F	0.446524	-1.609239	5.384946
F	2.614008	-1.835452	5.258281
F	3.687968	-2.086363	1.560839
C	4.259069	-0.994093	1.066800
F	4.561058	-0.159314	2.082179
S	5.850684	-1.397857	0.127473
N	5.315941	-2.380057	-1.042259
S	5.282690	-1.964191	-2.606947
C	7.076056	-2.137046	-3.179408
F	7.488856	-3.403580	-2.984733
O	6.333910	-0.087490	-0.347121
O	6.629560	-2.159934	1.103725
F	3.372216	-0.367601	0.267711
O	4.600331	-3.057103	-3.315221
O	4.898424	-0.578615	-2.899777
F	7.151939	-1.852801	-4.485558
F	7.885808	-1.315002	-2.502793
O	-5.196678	1.256426	-0.639897
S	-6.475836	1.035913	0.070939
O	-7.495104	2.079758	0.037796
N	-6.956101	-0.499157	-0.157009
S	-8.451777	-0.944974	-0.622326
C	-8.398590	-0.655141	-2.489525
F	-7.390461	-1.377780	-3.041515
O	-8.481021	-2.410721	-0.507959
O	-9.566141	-0.133450	-0.148490
C	-5.926491	0.879634	1.868430
F	-5.356785	2.033818	2.256698
F	-6.949320	0.601711	2.673515
F	-4.996450	-0.100303	1.989680
F	-9.546791	-1.038810	-3.054717
F	-8.178201	0.637412	-2.764491
H	6.470562	-5.761468	-1.390204
H	-3.557742	-6.055289	-2.638425
H	4.999826	4.358474	3.244492
H	-0.354402	-1.601192	-2.655981
H	-3.522165	-1.114869	-0.155205
H	-3.101938	0.081420	-1.361157
H	-1.086004	-1.099309	-0.647231
H	-1.759890	-2.668183	-1.063055
H	-1.028463	0.219632	-2.813376
H	-2.838595	1.436058	-3.164605
H	-1.756355	1.997621	-4.456955
H	-0.866196	3.762515	-2.875929
H	-2.542254	3.985632	-3.327295
H	0.920242	1.025652	-3.388296

H	2.470840	2.611136	-3.993354
H	3.691605	1.317392	-3.984745
H	3.386542	1.056974	-1.521655
H	2.218233	2.395895	-1.527692
H	1.626817	-0.850168	-2.702059
H	1.067045	-2.862328	-0.625558
H	2.721611	-2.519316	-1.137931
H	2.330538	-3.708306	-3.288877
H	0.625118	-3.934300	-2.864640
H	-3.406788	1.880883	0.431233
H	0.191049	0.265977	2.174501
H	1.493815	1.734381	0.606981
H	5.618555	1.671642	-0.066768
H	5.973998	2.225579	2.307028
H	3.658422	5.827100	1.690869
H	4.324683	-5.703042	-2.663936
H	6.432036	-5.223770	1.067569
H	2.160896	-4.740889	0.751758
H	-6.709711	-4.668566	-0.010913
H	-5.425333	-6.583746	-1.043277
H	-3.052961	-3.638281	-3.140015
H	4.178884	-4.651817	2.128222
H	-6.031647	-2.331192	-0.578390
H	3.332416	5.130644	-0.635063
H	-2.318713	0.391345	2.114717
H	0.235789	3.195793	-0.997373
H	1.999369	-6.017024	-2.529399
H	1.115519	-5.480880	-1.087286
H	-3.818932	-1.485002	-3.193229
H	-5.111606	-0.942787	-2.098752
H	-3.386548	3.205435	-1.195135
H	-2.154667	4.486079	-0.999714
H	4.013890	3.925045	-2.418464
H	5.186199	2.594315	-2.256907

6 Molecular Dynamics Simulations

SI Tab. S2: Lennard-Jones parameters σ and ϵ for all interaction sites of the $[HOC_2Py]^+$, $[HOC_3Py]^+$ and $[HOC_4Py]^+$ cation.

site	σ / Å	$\epsilon \cdot k_B^{-1}$ / K
N	3.25	85.55
C_a	3.55	35.23
H_a	2.42	15.10
C_c	3.50	33.20
H_c	2.50	15.10
H_m	2.50	15.10
O	3.12	85.60
H_o	0.00	0.00

SI Tab. S3: Bond length $r^0_{\kappa\lambda}$ and angle parameters $\phi^0_{\kappa\lambda\omega}$ und $k^a_{\kappa\lambda\omega}$ for the angle potential $V^a_{\kappa\lambda\omega} = \frac{1}{2}k^a_{\kappa\lambda\omega}(\phi_{\kappa\lambda\omega} - \phi^0_{\kappa\lambda\omega})^2$ in the force field of the $[HOC_2Py]^+$, $[HOC_3Py]^+$ and $[HOC_4Py]^+$ cation.

bond	$r^0_{\kappa\lambda}$ / Å	angle	$\phi^0_{\kappa\lambda\omega}$ / °	$k^a_{\kappa\lambda\omega}$ / kJ mol^{-1}rad^{-2}
C_a-N	1.339	C_a-C_a-C_a	120.0	527.20
C_a-H_a	1.080	C_a-C_a-N	124.0	585.80
C_a-C_a	1.400	C_a-N-C_a	117.0	585.80
N-C_c	1.339	C_a-C_a-H_a	120.0	292.90
C_c-C_c	1.529	N-C_a-H_a	116.0	292.90
C_c-H_c	1.090	C_a-N-C_c	121.5	585.80
C_c-O	1.410	N-C_c-C_c	112.7	487.43
O-H_o	0.945	H_c-C_c-N	110.7	313.26
C_c-H_m	1.090	H_c-C_c-H_c	107.8	275.70
		H_c-C_c-C_c	110.7	313.26
		H_m-C_c-C_c	110.7	313.26
		H_m-C_c-H_m	107.8	275.70
		C_c-C_c-C_c	112.7	487.43
		H_o-O-C_c	108.5	460.55
		C_c-C_c-O	109.5	418.68
		H_c-C_c-O	109.5	293.08

SI Tab. S4: Parameters m_n, k_m^{dp} and ψ_m^0 for the improper dihedral potential $V_{\kappa\lambda\omega\tau}^{\mathrm{dp}} = \sum_n k_m^{\mathrm{dp}}[1 + \cos(m_n\psi_m - \psi_m^0)]$ in the force field of the $[HOC_2Py]^+$, $[HOC_3Py]^+$ and $[HOC_4Py]^+$ cation. The central atom is the first in the list.

	m_n	k_m^{dp} / kJ mol^{-1}	ψ_m^0 / °
N-C_a-C_a-C_c	2	4.6060	180.0
C_a-N-C_a-H_a	2	4.6060	180.0
C_a-C_a-C_a-H_a	2	4.6060	180.0

SI Tab. S5: Parameters m_n, k_m^{dp} and ψ_m^0 for the torsion potential $V_{\kappa\lambda\omega\tau}^{\mathrm{dp}} = \sum_n k_m^{\mathrm{dp}}[1+\cos(m_n\psi_m - \psi_m^0)]$ in the force field of the $[HOC_2Py]^+$ cation.

	$n(\kappa\lambda\omega\tau)$	m_n	k_m^{dp} / kJ mol^{-1}	ψ_m^0 / °
X-C_a-C_a-X	1	2	15.1780	180.0
X-C_a-N-X	1	2	15.1780	180.0
C_a-N-C_c-C_c	1	2	0.0802	0
	2	4	-0.4693	0
N-C_c-C_c-O	1	1	-0.7375	0.0
	2	2	1.8576	0.0
	3	3	7.2898	0.0
C_c-C_c-O-H_o	1	1	-5.8097	0.0
	2	2	1.8939	0.0
	3	3	2.5150	0.0

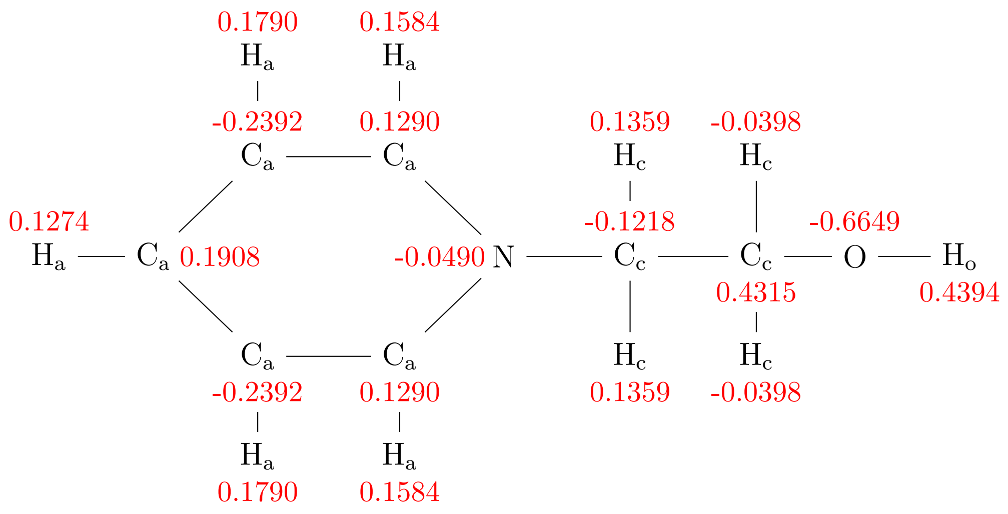

SI Fig. S7: Structure of the $[HOC_2Py]^+$ cation with atom types and corresponding point charges q/e in red.

SI Tab. S6: Parameters m_n, k_m^{dp} and ψ_m^0 for the torsion potential $V_{\kappa\lambda\omega\tau}^{dp} = \sum_n k_m^{dp}[1+\cos(m_n\psi_m - \psi_m^0)]$ in the force field of the $[HOC_3Py]^+$ cation.

	$n(\kappa\lambda\omega\tau)$	m_n	k_m^{dp} / kJ mol^{-1}	ψ_m^0 / °
X-C_a-C_a-X	1	2	15.1780	180.0
X-C_a-N-X	1	2	15.1780	180.0
C_a-N-C_c-C_c	1	2	-0.7379	0
	2	4	-0.2237	0
N-C_c-C_c-C_c	1	1	-3.0206	0.0
	2	2	0.6685	0.0
	3	3	5.1206	0.0
	4	4	0.4708	0.0
C_c-C_c-C_c-O	1	1	4.1980	0.0
	2	3	5.2448	0.0
	3	4	1.0859	0.0
C_c-C_c-O-H_o	1	1	-1.8926	0.0
	2	2	1.0349	0.0
	3	3	2.5840	0.0
	4	4	0.0316	0.0

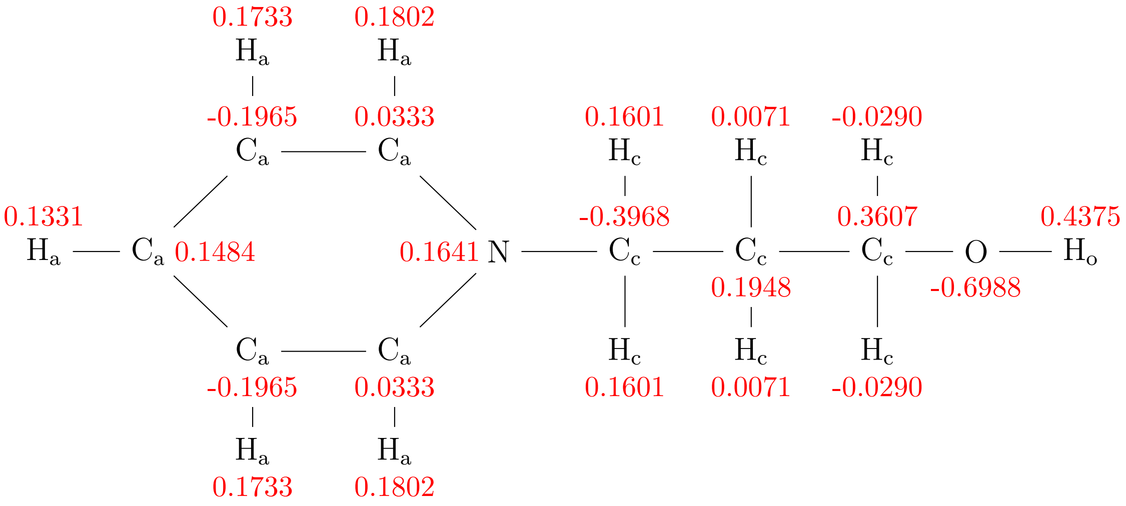

SI Fig. S8: Structure of the $[HOC_3Py]^+$ cation with atom types and corresponding point charges q/e in red.

SI Tab. S7: Parameters m_n, k_m^{dp} and ψ_m^0 for the torsion potential $V_{\kappa\lambda\omega\tau}^{dp} = \sum_n k_m^{dp}[1+\cos(m_n\psi_m - \psi_m^0)]$ in the force field of the $[HOC_4Py]^+$ cation.

	$n(\kappa\lambda\omega\tau)$	m_n	k_m^{dp} / kJ mol^{-1}	ψ_m^0 / °
X-C$_a$-C$_a$-X	1	2	15.1780	180.0
X-C$_a$-N-X	1	2	15.1780	180.0
C$_a$-N-C$_c$-C$_c$	1	2	-0.3579	0
C$_a$-N-C$_c$-C$_c$	2	4	-0.4037	0
C$_c$-C$_c$-C$_c$-C$_c$	1	1	-0.2825	0.0
	2	2	0.6065	0.0
	3	3	4.6858	0.0
	4	4	0.7018	0.0
	5	5	0.4468	0.0
	6	6	0.4564	0.0
C$_c$-C$_c$-C$_c$-O	1	1	-2.3748	0.0
	2	3	6.8089	0.0
	3	4	0.9531	0.0
C$_c$-C$_c$-O-H$_o$	1	1	-3.5552	0.0
	2	2	0.5886	0.0
	3	3	2.5272	0.0
	4	4	0.1504	0.0

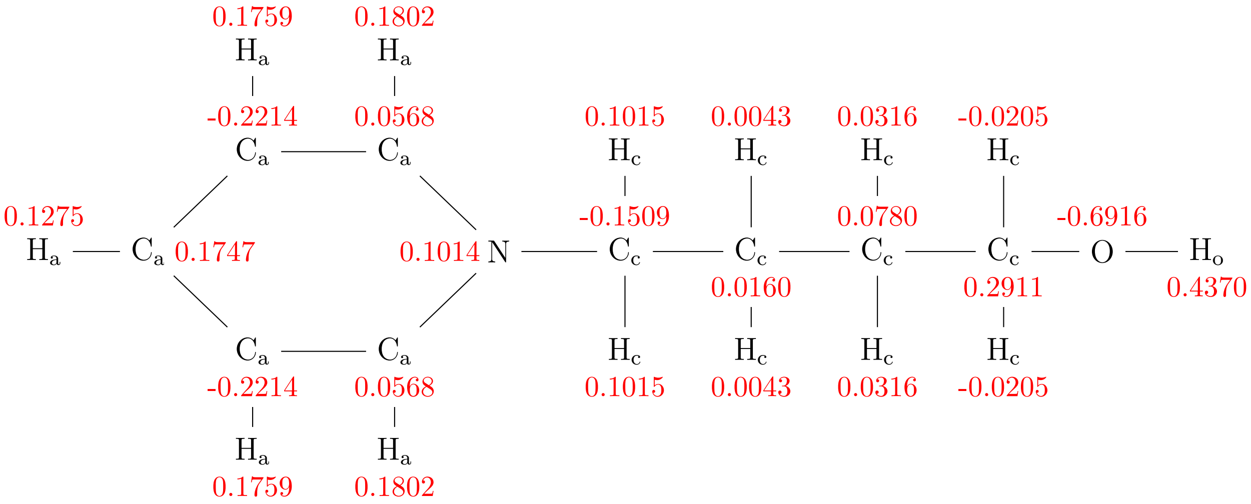

SI Fig. S9: Structure of the $[HOC_4Py]^+$ cation with atom types and corresponding point charges in red.
